# Bacteriophage-Based Control of Methicillin-Resistant *Staphylococcus aureus*: Anti-Biofilm Activity, Surface-Active Formulation Compatibility, and Genomic Context

**DOI:** 10.3390/antibiotics15020155

**Published:** 2026-02-02

**Authors:** Peechanika Chopjitt, Wanwisa Kanha, Achiraya Sachit, Juthamas Thongkam, Phinkan Kanthain, Pornnapa Pradabsri, Supreeya Paiboon, Sirinan Thananchai, Surasak Khankhum, Anusak Kerdsin, Nuchsupha Sunthamala

**Affiliations:** 1 Faculty of Public Health, Kasetsart University Chalermphrakiat Sakon Nakhon Campus, Sakon Nakhon 47000, Thailand; peechanika.c@ku.th (P.C.); anusak.ke@ku.th (A.K.); 2 Department of Biology, Faculty of Science, Mahasarakham University, Mahasarakham 44150, Thailandsurasak.kk@msu.ac.th (S.K.); 3 Epidemic Simulation and Aetiology Nexus for Infectious Diseases, Mahasarakham University, Mahasarakham 44150, Thailand

**Keywords:** surface decontamination, healthcare-associated pathogens, one health antimicrobial strategies, non-antibiotic antimicrobials, infection prevention and control

## Abstract

Background/Objectives: Methicillin-resistant *Staphylococcus aureus* (MRSA) continues to pose a significant challenge for infection prevention, particularly because of its ability to persist on surfaces and form resilient biofilms. Although bacteriophages have attracted renewed interest as alternatives or complements to chemical disinfectants, their applied use requires careful assessment of antimicrobial performance, formulation tolerance, and genomic context. Methods: Staphylococcus-infecting bacteriophages were isolated from environmental sources and examined against reference *Staphylococcus* isolates. Two phage isolates, designated MRSA-W3 and SA-W2, displayed lytic activity against a broad subset of clinical MRSA strains. Using a time-resolved agar-based infection assay, phage exposure resulted in a multiplicity-of-infection-dependent decline in viable MRSA populations. Results: Time-resolved infection assays revealed a multiplicity-of-infection-dependent reduction in viable MRSA, with a pronounced decrease observed approximately 40 min post-infection. At this time point, phage-treated cultures showed a reduction of 1.2–1.8 log_10_ CFU/mL relative to untreated controls (mean Δlog_10_ = 1.5; 95% CI, 1.1–1.9), while control cultures remained stable. Quantitative biofilm assays demonstrated that both phages reduced biofilm biomass compared with untreated conditions, with inhibition values ranging from 20% to 45% across isolates (*p* ≤ 0.05), reflecting strain-dependent but reproducible effects. Assessment of formulation compatibility indicated that both phages retained infectivity following exposure to sodium dodecyl sulfate, Triton X-100, and Tween 80, whereas ethanol (≥10%) and higher concentrations of dimethyl sulfoxide were associated with rapid loss of activity. In surface disinfection models, selected phage–surfactant formulations achieved a maximum reduction of 2.18 log_10_ CFU/cm^2^ compared with untreated controls (*p* ≤ 0.05). Infection-coupled whole-genome sequencing of MRSA-infecting phage MRSA-W3 produced a high-quality assembly (99.99% completeness; 0.13% contamination) and revealed a mosaic genome containing incomplete prophage-like regions, which were interpreted conservatively as evidence of shared phage ancestry rather than active temperate behavior. Conclusions: Therefore, these findings suggest that bacteriophage-based approaches may be feasible for MRSA surface decontamination, while clearly emphasizing the need for context-specific validation before practical implementation.

## 1. Introduction

Methicillin-resistant *Staphylococcus aureus* (MRSA) remains a leading cause of healthcare-associated infections and continues to challenge infection prevention and control (IPC) programs worldwide [1,2]. In addition to its resistance to multiple antibiotic classes, MRSA exhibits a pronounced ability to survive on abiotic surfaces and to form biofilms, enabling prolonged environmental persistence and facilitating indirect transmission within clinical settings [3,4,5]. Environmental reservoirs, particularly frequently touched hospital surfaces, have therefore been recognized as critical contributors to MRSA dissemination, underscoring the importance of effective surface decontamination strategies alongside patient-centered interventions [6,7].

Chemical disinfectants remain the cornerstone of surface hygiene in healthcare environments; however, their real-world performance can be influenced by formulation stability, organic load, surface material, and repeated application [7,8]. Moreover, increasing attention has been directed toward the unintended consequences of intensive biocide use, including material degradation, occupational exposure risks, and the potential selection of disinfectant-tolerant microbial populations [9,10,11]. These limitations have stimulated growing interest in complementary, non-antibiotic antimicrobial approaches that can be integrated into existing IPC frameworks rather than replacing them outright [12].

Bacteriophages, viruses that specifically infect bacteria, have re-emerged as promising candidates in this context due to their host specificity, self-propagating nature, and activity against antibiotic-resistant pathogens [13,14,15]. While phage therapy for clinical infections has gained renewed momentum, considerably less attention has been paid to the systematic evaluation of bacteriophages for environmental or surface disinfection applications [16,17]. Importantly, translation into applied settings requires evidence beyond planktonic lytic activity, including efficacy against biofilm-associated cells, stability in the presence of surfactants or solvents used in disinfectant formulations, and reproducible reductions in recoverable bacteria on surfaces under controlled conditions [18,19,20].

Recent studies have also emphasized the importance of genomic characterization in phage-based applications, particularly to contextualize safety, stability, and evolutionary relationships [21,22,23,24]. Many *Staphylococcus*-infecting phages exhibit mosaic genome architectures shaped by horizontal gene transfer, and the detection of prophage-like regions must be interpreted cautiously, as sequence similarity alone does not establish functional temperate behavior or inducibility [25,26]. Integrating genomic data with phenotypic assays therefore provides a more robust framework for evaluating phage suitability for applied antimicrobial use.

In the present study, we isolated *Staphylococcus*-infecting bacteriophages from environmental samples and evaluated their activity against a diverse panel of clinical and reference *Staphylococcus* isolates. Using time-resolved infection assays, quantitative biofilm inhibition analyses, and surface disinfection models, we assessed phage-mediated reductions in viable MRSA under conditions relevant to environmental decontamination. In parallel, we examined phage compatibility with commonly used surfactants and solvents to inform formulation feasibility. Finally, infection-coupled metagenomic assembly was performed to characterize the genomic context of a representative phage, with conservative interpretation of prophage-like regions within assembled contigs. Consistent with emerging guidance in applied phage research, genomic features identified in this study are interpreted as contextual markers of evolutionary relatedness rather than as definitive indicators of temperate behavior, and the findings are framed to inform controlled, surface-based applications rather than direct clinical use. Together, these data aim to inform the practical potential and limitations of bacteriophage-based formulations as adjunct tools for MRSA surface decontamination within contemporary IPC strategies.

## 2. Materials and Methods

### 2.1. Preparation of Bacterial Host Strains

*Staphylococcus aureus* (MRSA) DMST 20654, *S. aureus* TISTR 746, and *Staphylococcus epidermidis* TISTR 518 were retrieved from glycerol stocks and cultured in Nutrient Broth at 37 °C for 18–24 h. Cultures were streaked onto Nutrient Agar (Himedia, Mumbai, India) and Mannitol Salt Agar (Himedia, Mumbai, India) to confirm colony morphology. Gram staining and microscopic examination were performed to verify bacterial identity prior to use as host strains. Media were selected based on assay-specific requirements and standard protocols [1,27]. All experimental procedures involving bacterial pathogens and bacteriophages were conducted in accordance with institutional biosafety guidelines and applicable national regulations. This study was reviewed and approved by the Institutional Biosafety Committee (IBC), Mahasarakham University, under approval number IBC006-004/2568. All laboratory work was performed in certified biosafety facilities by trained personnel, with appropriate containment and waste disposal procedures to ensure biological safety.

### 2.2. Isolation of Bacteriophages from Environmental Samples

Environmental water (20 mL) and soil (20 g) samples were mixed in sterile 50 mL tubes and allowed to sediment. The aqueous phase was centrifuged at 4500× *g* for 10 min at 4 °C. Supernatants were inoculated into Brain Heart Infusion (BHI) broth (Himedia, Mumbai, India) (20 mL) containing the respective host bacteria and incubated at 37 °C for 24 h. Enriched samples were centrifuged at 4500× *g* for 30 min at 4 °C, filtered through 0.2 µm syringe filters (Whatman, Leeds, UK), aliquoted (10 mL), and stored at 4 °C until further analysis [28,29].

### 2.3. Detection of Bacteriophages by Spot Test

Host bacteria were grown to mid-log phase by subculturing (1%, *v*/*v*) in Nutrient Broth (Himedia, Mumbai, India) and incubating at 37 °C with shaking (200 rpm) for 2 h. Bacterial suspensions were adjusted to 0.5 McFarland using 1× PBS. Aliquots (1 mL) were spread onto Nutrient Agar (Himedia, Mumbai, India) supplemented with 0.1% (*w*/*v*) CaCl_2_ (Sigma-Aldrich, Burlington, MA, USA). After drying, 10 µL drops of filtered phage lysates were applied to the agar surface and incubated at 37 °C for 18–24 h. Plaque formation was recorded, and single plaques were excised and suspended in SM buffer (Thermo Fisher Scientific, Waltham, MA, USA) [28,29].

### 2.4. Phage Purification by Drop Plate Method

Single-plaque purification was performed by repeated drop plate assays. Briefly, host bacteria were prepared as described above and overlaid onto Nutrient Agar (Himedia, Mumbai, India) containing 0.1% (*w*/*v*) CaCl_2_ (Sigma-Aldrich, MA, USA). Phage suspensions (10 µL per drop) were applied, incubated at 37 °C for 24 h, and individual plaques were recovered into SM buffer (Thermo Fisher Scientific, MA, USA). This purification step was repeated three times to ensure clonal phage populations [28,29].

### 2.5. Host Range Screening by Spot Test

The host range of bacteriophages MRSA-W3 and SA-W2 was evaluated using a spot-test screening assay against a panel of *Staphylococcus* and non-*Staphylococcus* bacterial strains. Test bacteria were cultured in Tryptic Soy Broth (Himedia, Mumbai, India) at 37 °C for 18–24 h, subcultured at 1% (*v*/*v*), and incubated with shaking (200 rpm) for 2 h to obtain exponentially growing cells. Bacterial suspensions were adjusted to 0.5 McFarland using sterile phosphate-buffered saline (Sigma-Aldrich, MA, USA). Standardized bacterial lawns were prepared by spreading 1.0 mL of the suspension onto tryptic soy agar (Himedia, Mumbai, India) supplemented with 0.1% (*w*/*v*) CaCl_2_ (Sigma-Aldrich, MA, USA), followed by air-drying. Phage suspensions were serially diluted in SM buffer (Thermo Fisher Scientific, MA, USA) (10^5^–10^7^ PFU/mL), and 10 µL of each dilution was spotted onto the bacterial lawns. Plates were incubated at 37 °C for 18–24 h and examined for zones of clearing. Spot-test results were recorded as clear, turbid, or absent lysis. Observed clearing was interpreted as growth inhibition only and not as evidence of productive infection, particularly for non-*Staphylococcus* species. Productive infection was considered confirmed only for *Staphylococcus* hosts supporting plaque formation and serial propagation. Non-*Staphylococcus* species (e.g., *Escherichia coli*, *Pseudomonas aeruginosa*, *Acinetobacter baumannii*, *Salmonella Typhimurium*) are now explicitly described as specificity controls, included to assess the predominant staphylococcal tropism of the isolated phages [28,29].

### 2.6. Evaluation of Phage Stability Under Temperature Stress

Purified phages (10^7^ PFU/mL) were incubated at 25, 37, 50, and 60 °C for 5, 30, 60, 120, and 180 min. At each time point, phage suspensions were mixed with molten soft agar (TSB + 0.5% agar) (Himedia, Mumbai, India) and poured onto Tryptic Soy Agar (Himedia, Mumbai, India) plates supplemented with 0.1% (*w*/*v*) CaCl_2_ (Sigma-Aldrich, MA, USA). After incubation at 37 °C for 18–24 h, plaque-forming units (PFU/mL) were enumerated [30,31].

### 2.7. Stability of Bacteriophages in Surfactants and Organic Solvents

Phage suspensions were exposed to surfactants including sodium dodecyl sulfate (SDS) (Sigma-Aldrich, MA, USA), Tween 80 (Sigma-Aldrich, MA, USA), and Triton X-100 (Sigma-Aldrich, MA, USA), and organic solvents including ethanol (Sigma-Aldrich, MA, USA) and dimethyl sulfoxide (DMSO) (Sigma-Aldrich, MA, USA), at final concentrations of 0.1%, 1%, 5%, 10%, 20%, and 35% (*v*/*v* or *w*/*v*, as appropriate) for 1 h at room temperature. Treated phages were serially diluted (10^9^–10^4^ PFU/mL) and assessed for lytic activity by spot test on TSA plates (Himedia, Mumbai, India) supplemented with 0.1% (*w*/*v*) CaCl_2_ (Sigma-Aldrich, MA, USA). A fixed working dilution (10^−3^) was selected based on preliminary screening to ensure consistent lytic activity across assays. Phage suspensions were applied at a defined initial working concentration (10^7^ PFU/mL). However, post-exposure absolute PFU/mL quantification was not performed for formulation compatibility and surface efficacy assays, which were designed to assess comparative lytic performance under defined conditions rather than detailed phage kinetic parameters [31].

### 2.8. Glass-Based Container Surfaces Disinfection Assay

Glass-based container surfaces were inoculated with host bacteria adjusted to 0.5 McFarland and allowed to dry. Phage–surfactant-based formulations (10^7^ PFU/mL, 100 µL) were applied to the contaminated surfaces. At 2, 5, 10, 15, and 20 min, surfaces were swabbed with sterile cotton swabs and suspended in 0.85% NaCl (Sigma-Aldrich, MA, USA). Aliquots (100 µL) were spread onto TSA plates (Himedia, Mumbai, India) and incubated at 37 °C for 18–24 h. Viable bacteria were quantified as CFU/mL, and results were expressed as log_10_ reductions relative to untreated controls [30,32,33,34,35,36,37]. Δlog_10_ reduction was calculated as: Δlog_10_ = log_10_(CFUcontrol) − log_10_(CFUtreated). Accordingly, the surface experiment is presented as a carrier-like surface model (carrier-like assay) intended to assess adjunct-level phage activity under defined laboratory conditions, rather than as a standardized EN or ASTM disinfectant efficacy test. Chemical neutralizers were not applied following surface exposure because phage inactivation was assessed by physical recovery and dilution, and residual surfactant concentrations were below levels shown to inhibit bacterial regrowth in control experiments. Unexposed surface controls and phage-free surfactant controls were included to account for background variability and residual formulation effects.

### 2.9. Evaluation of Anti-Biofilm Activity

Biofilm inhibition assays were performed using a crystal violet staining method. Host bacteria were cultured in tryptic soy broth (Himedia, Mumbai, India) at 37 °C for 18–24 h and adjusted to 0.5 McFarland (10^8^ CFU/mL) using TSB (Himedia, Mumbai, India supplemented with 10 mM MgSO_4_ (Sigma-Aldrich, MA, USA).

Bacterial suspensions (100 µL) were dispensed into 96-well microplates (Thermo Fisher Scientific, MA, USA) (three technical replicates per isolate) and incubated at 37 °C for 48 h. Phage suspensions (10^7^ PFU/mL; 100 µL) were then added to each well, while untreated wells served as negative controls. Plates were further incubated at 37 °C for 72 h. After incubation, wells were gently washed twice with deionized water, stained with 0.1% crystal violet (Sigma-Aldrich, MA, USA) for 10 min, and rinsed three times. Bound dye was solubilized with 150 µL ethanol (Sigma-Aldrich, MA, USA), and absorbance was measured at 595 nm using an AMR-100 microplate reader (Biobase, Jinan, China). Biofilm inhibition was calculated relative to untreated controls [30,38,39]. Extended incubation was used to evaluate phage-associated effects on established biofilms, reflecting conditions relevant to surface persistence rather than early biofilm development.

### 2.10. Agar-Based Phage Infection Assay with Time-Resolved CFU Enumeration

Due to challenges associated with bacteriophage propagation and purification, an infection-coupled whole-genome sequencing approach was employed to identify the bacteriophage MRSA-W3. Methicillin-resistant *Staphylococcus aureus* (MRSA) strains were cultivated in nutrient broth (NB) (Himedia, Mumbai, India) at 37 °C for 24 h. An aliquot (1%, *v*/*v*) of the overnight culture was subcultured into fresh NB and incubated at 37 °C with shaking at 200 rpm for 2 h to obtain exponentially growing cells. The bacterial suspension was adjusted to approximately 10^7^ CFU/mL prior to infection. For phage infection, 200 µL of the bacterial suspension was transferred into sterile microcentrifuge tubes, followed by the addition of 50 µL of CaCl_2_ solution (Sigma-Aldrich, MA, USA) (final concentration 2 mM) and 50 µL of bacteriophage suspension at the indicated multiplicity of infection (MOI). The mixture was gently mixed and incubated at room temperature for 15 min to allow phage adsorption. After adsorption, the infection mixtures were incubated at 37 °C. At defined time points (every 10 min for up to 2 h), aliquots were withdrawn, serially diluted in sterile phosphate-buffered saline (PBS) (Sigma-Aldrich, MA, USA), and plated onto nutrient agar plates supplemented with CaCl_2_ (Sigma-Aldrich, MA, USA). Plates were incubated at 37 °C for 18–24 h, after which bacterial colonies were enumerated. Viable bacterial counts were expressed as CFU/mL, and reductions in bacterial viability over time were used to assess the kinetics of phage infection and bacterial killing. Uninfected bacterial cultures treated identically but without phage served as negative controls [40]. MOI values reported in kinetic experiments correspond to the stated PFU/mL relative to ~10^7^ CFU/mL input.

### 2.11. Bacterial DNA Extraction and Genome Analysis

Genomic DNA was extracted from *Staphylococcus aureus* cultures following infection with bacteriophage MRSA-W3 using a phenol–chloroform extraction protocol (Sigma-Aldrich, MA, USA) optimized for double-stranded DNA bacteriophages. Briefly, infected cultures were treated with proteinase K (Vivantis Technologies, Selangor, Malaysia) and sodium dodecyl sulfate (SDS) (Sigma-Aldrich, MA, USA), followed by sequential phenol–chloroform–isoamyl alcohol extraction (Sigma-Aldrich, MA, USA) and ethanol (Sigma-Aldrich, MA, USA) precipitation. DNA concentration and purity were assessed spectrophotometrically prior to sequencing [41]. Paired-end whole-genome sequencing was performed on an Illumina platform (Illumina, San Diego, CA, USA). Raw sequencing reads were subjected to quality control and adapter trimming using an automated pipeline, and high-quality reads were assembled de novo using SPAdes (v3.15). Assembly quality metrics, including total assembly size, contig number, N50, L50, and GC content, were evaluated using QUAST. Genome completeness and contamination were estimated using a machine-learning–based approach. Genome annotation was conducted with Prokka using translation table. Taxonomic profiling of sequencing reads was performed using multiple complementary strategies, including k-mer–based classification, read mapping, and alignment-based approaches, to increase robustness at the strain level. Viral reads were further compared against curated bacteriophage reference databases to identify closely related phage lineages. Prophage-like regions within the assembled genome were identified using PHASTER, based on sequence similarity to known phage proteins and the presence of hallmark phage genes such as capsid, terminase, integrase, tail, and portal proteins. Detected regions were classified as intact, questionable, or incomplete according to PHASTER scoring criteria and were visualized at the contig level. Importantly, the identified prophage-like regions were consistently classified as incomplete and lacked experimental evidence of inducibility; therefore, these features were interpreted conservatively as reflecting mosaic genomic ancestry rather than functional temperate behavior [42,43,44,45,46,47,48,49,50,51,52,53,54,55,56,57,58,59,60].

### 2.12. Statistical Analysis

All experiments were conducted in at least triplicate. Quantitative data are presented as mean ± standard deviation (SD). Differences among treatment groups were evaluated using one-way or two-way analysis of variance (ANOVA), as appropriate, followed by Tukey’s multiple comparison post hoc test. All surface disinfection experiments were performed using independent biological replicates, and results are reported as mean Δlog_10_ CFU/cm^2^ ± standard deviation (or 95% confidence intervals) [61]. Log_10_ reductions in bacterial counts were calculated relative to untreated controls. A *p*-value ≤ 0.05 was considered statistically significant. Statistical analyses were performed using GraphPad Prism version 10 (GraphPad Software, Boston, MA, USA) [62,63].

## 3. Results

### 3.1. Isolation and Phenotypic Characterization of Staphylococcus Host Strains

**Figure 1:**
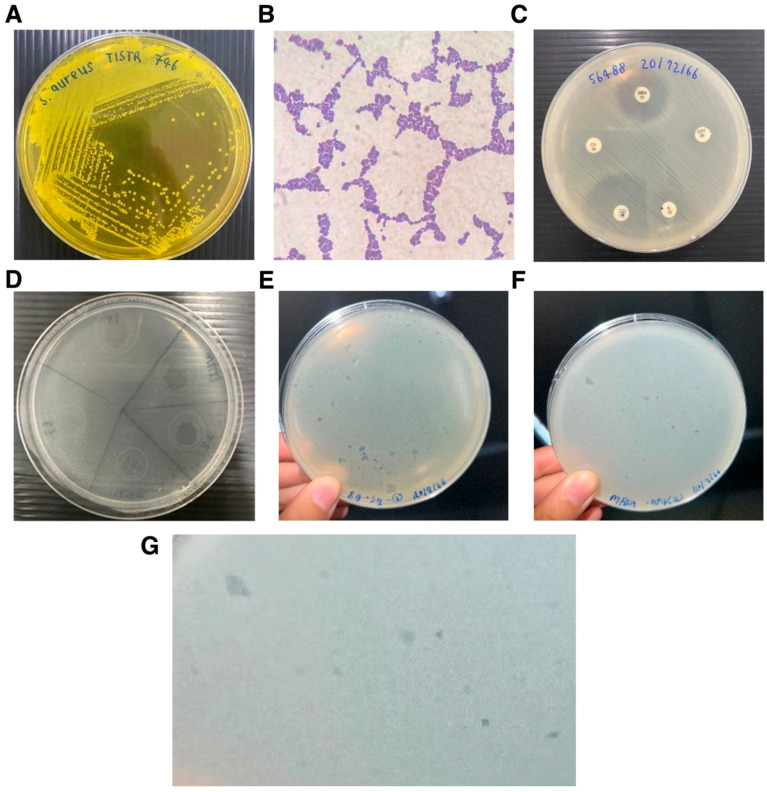
Isolation, characterization, and infection dynamics of methicillin-resistant *Staphylococcus aureus* (MRSA) and its corresponding bacteriophage. (**A**) Representative colonies of *Staphylococcus aureus* exhibiting mannitol fermentation on mannitol salt agar (MSA). (**B**) Cellular morphology of *S. aureus* observed by Gram staining, demonstrating Gram-positive cocci arranged in clusters. (**C**) Antimicrobial susceptibility testing of *S. aureus* isolates performed by disk diffusion, showing resistance to cefoxitin (30 µg), consistent with methicillin resistance, and representative results of the D-zone test indicating inducible clindamycin resistance, interpreted according to CLSI guidelines. (**D**) Representative spot test assay used for screening bacteriophages during the isolation process. (**E**,**F**) Representative images illustrating the double-layer agar (agar overlay) method used for bacteriophage propagation and plaque detection. (**G**) Plaque morphology of bacteriophages isolated from soil and water samples, showing distinct plaque phenotypes.

Host strains for bacteriophage isolation and characterization, comprising reference strains (*S. aureus* [MRSA] DMST 20654, *S. aureus* TISTR 746, and *S. epidermidis* TISTR 518). Fifty clinical *S. aureus* isolates obtained from diverse specimen sources, predominantly blood, as well as sputum, pus, wound, tissue, urine, and cerebrospinal fluid were used for host range screening by spot test (Table S1). Phenotypic characterization confirmed that all clinical *S. aureus* isolates were Gram-positive cocci, catalase-positive, oxidase-negative, DNase-positive, and capable of mannitol fermentation, consistent with typical *S. aureus* profiles. In contrast, *S. epidermidis* TISTR 518 was catalase-positive but DNase-negative and non-fermentative on mannitol salt agar, supporting species-level differentiation (Figure 1A–C and Table S1). Antimicrobial susceptibility testing revealed considerable heterogeneity among the clinical isolates. Most exhibited resistance to cefoxitin, confirming MRSA status, while variable susceptibility was observed for cotrimoxazole, gentamicin, clindamycin, and erythromycin, with several isolates displaying multidrug-resistant phenotypes (Table S1). Reference strains showed antibiotic profiles consistent with their documented characteristics.

Overall, the phenotypic and antimicrobial diversity of the host strain panel provided a clinically relevant and robust foundation for bacteriophage isolation, host-range analysis, and subsequent evaluation of phage activity against planktonic and biofilm-associated *Staphylococcus* cells.

### 3.2. Environmental Isolation and Detection of Staphylococcus-Infecting Bacteriophages

Bacteriophages capable of infecting *Staphylococcus* spp. were isolated from soil and water samples collected from cattle sheds and surrounding environments (Table 1). Initial detection using spot test assays revealed clear lytic zones against *S. aureus* reference strains (Figure 1D). Water-derived samples generally yielded broader lytic activity than soil-derived samples. Subsequent propagation using double-layer agar methods produced plaques with distinct morphologies, suggesting the presence of multiple phage types (Figure 1E–G). Nine representative phages were selected for further characterization based on plaque clarity and reproducibility.

### 3.3. Host Range of Isolated Bacteriophages Against Clinical S. aureus Isolates

**Figure 2:**
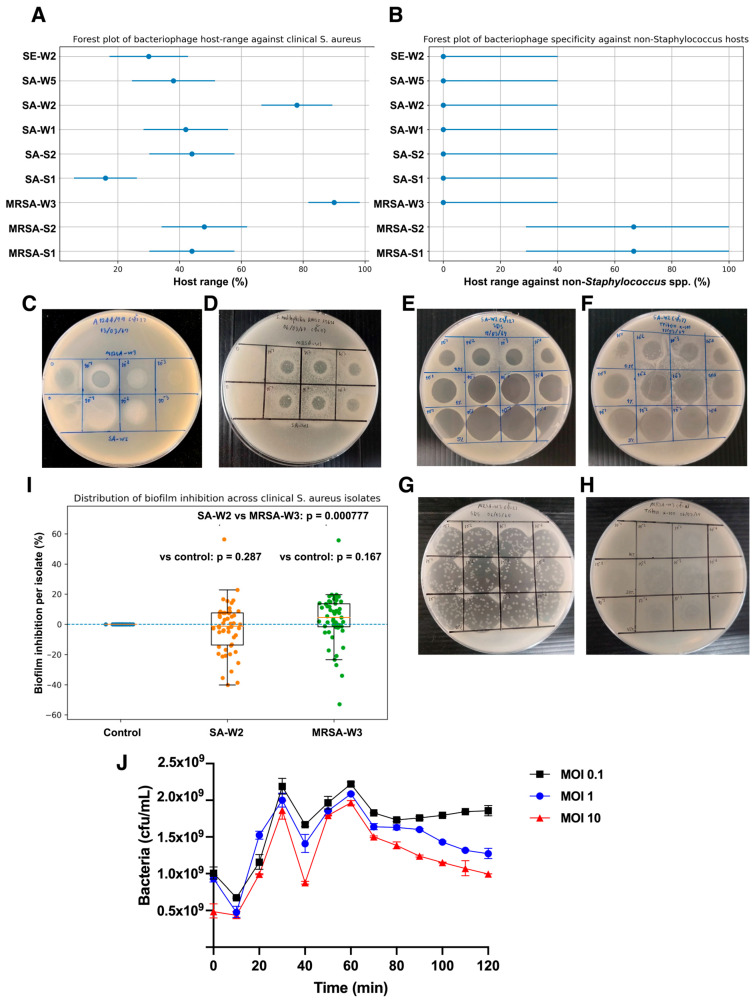
Host range, lytic activity, stability, and anti-biofilm assays of bacteriophages MRSA-W3 and SA-W2. (**A**) Forest plot depicting host-range assessment of bacteriophages against clinical *Staphylococcus aureus* isolates. Points represent the proportion of susceptible strains, and horizontal bars indicate 95% confidence intervals calculated using a binomial distribution (*n* = 50 strains per phage). (**B**) Host-range evaluation against non-*Staphylococcus* bacterial species determined by spot test assays, presented as binary outcomes (plaques observed or not observed). (**C**) Representative images of spot test assays showing lysis patterns of bacteriophages against heterologous *S. aureus* strains at different phage dilutions. (**D**) Representative images of spot test assays used to assess cross-genus infectivity of bacteriophages against non-*Staphylococcus* bacterial species at different dilutions. Representative images illustrating bacteriophage stability following exposure to surfactant solutions. Stability assays of bacteriophage SA-W2 following exposure to (**E**) SDS and (**F**) Triton X-100. Stability assays of bacteriophage MRSA-W3 following exposure to (**G**) SDS and (**H**) Triton X-100. (**I**) Boxplots showing the distribution of biofilm inhibition (%) among clinical *S. aureus* isolates following treatment with bacteriophages MRSA-W3 and SA-W2. Boxes represent the median and interquartile range, and dots indicate individual isolates. The orange line represents the median percentage of biofilm inhibition observed across clinical *Staphylococcus aureus* isolates for each bacteriophage treatment. Statistical significance was determined using one-way ANOVA followed by Tukey’s multiple-comparison test. (**J**) Time-resolved bacterial viability during MRSA-W3 infection of MRSA measured by agar-based CFU enumeration. Viable counts were recorded at 10 min intervals over a 120 min incubation period.

The host range of nine bacteriophages was evaluated against 50 clinical *S. aureus* isolates using spot test assays (Figure 2A, Table 2 and Table S2). Among the tested phages, MRSA-W3 exhibited the broadest host range, infecting 39 of 50 isolates (78.0%; 95% CI: 64.8–87.3), followed by SA-W2 (58.0%; 95% CI: 44.2–70.7) and MRSA-S2 (50.0%; 95% CI: 36.8–63.2). The remaining phages exhibited more restricted activity, with spot-test positivity ranging from 28.0% to 44.0% of isolates. When evaluated against non-*Staphylococcus* species, spot-test clearing was infrequently observed and was limited to MRSA-W3 and SA-W2, which produced zones of growth inhibition on five of seven non-*Staphylococcus* strains tested (71.4%; 95% CI: 35.9–91.8) (Figure 2B; Table 2 and Table S2). Importantly, these observations were restricted to spot-test clearing only and did not demonstrate productive infection, as no plaque formation, serial propagation, or efficiency-of-plating assays were observed on non-*Staphylococcus* hosts. Accordingly, these effects are interpreted conservatively as non-productive growth inhibition rather than true cross-genus infectivity. Overall, the spot-test data identify MRSA-W3 as the most broadly active phage within *S. aureus* hosts, while supporting a predominantly staphylococcal tropism for the phages examined.

### 3.4. Anti-Biofilm Activity of Bacteriophages MRSA-W3 and SA-W2

The anti-biofilm activity of bacteriophages MRSA-W3 and SA-W2 was quantitatively evaluated against a panel of clinical *Staphylococcus aureus* isolates using a crystal violet–based biofilm assay (Figure 2I and Table S3). Untreated control cultures displayed substantial inter-isolate variability in biofilm biomass, reflecting the heterogeneous biofilm-forming capacity of clinical strains. Treatment with SA-W2 resulted in a consistent shift toward reduced biofilm biomass relative to controls. Across all tested isolates, SA-W2 achieved a median biofilm reduction of approximately 28%, with an estimated 95% confidence interval (CI) of 22–38%. While several isolates exhibited modest inhibition (<15%), others showed reductions exceeding 40%, indicating isolate-dependent susceptibility rather than uniform biofilm suppression. MRSA-W3 demonstrated a slightly greater overall anti-biofilm effect. The median biofilm reduction was approximately 35%, with an estimated 95% CI of 25–45%, and a broader upper range of inhibition values compared with SA-W2. For a subset of isolates, MRSA-W3 reduced biofilm biomass by more than 45%, whereas a limited number of strains showed minimal response, underscoring heterogeneity in phage–host interactions.

Statistical analysis confirmed that the median biofilm inhibition for both MRSA-W3 and SA-W2 was significantly greater than zero (*p* ≤ 0.05), demonstrating a reproducible anti-biofilm effect across the isolate panel. In contrast, non-*Staphylococcus* control strains exhibited minimal or no reduction in biofilm biomass, supporting the host specificity of the observed activity. Collectively, these results indicate that both bacteriophages exert moderate but statistically significant biofilm inhibition, with MRSA-W3 showing a trend toward higher median efficacy. The observed variability among isolates highlights the importance of strain-level evaluation when considering bacteriophages for anti-biofilm or surface decontamination applications. Crystal violet staining was used to assess net biofilm biomass following prolonged phage exposure and should be interpreted as a phenotypic measure of biofilm inhibition rather than a direct indicator of phage replication or biofilm clearance.

### 3.5. Thermal Stability of Bacteriophages MRSA-W3 and SA-W2

Thermal stability assays showed that MRSA-W3 retained plaque-forming ability across a wide temperature range (25–60 °C) and exposure times up to 180 min, with detectable plaques across all phage concentrations (10^9^–10^4^ PFU/mL) (Table 3 and Table S4a). In contrast, SA-W2 exhibited partial thermal sensitivity at 37 °C, with loss of detectable plaques at 10^4^ PFU/mL, while remaining stable at lower dilutions and at elevated temperatures (50–60 °C) (Table 3 and Table S4b). These findings indicate greater thermal robustness of MRSA-W3 relative to SA-W2.

### 3.6. Time-Resolved Bacterial Viability During Phage Infection

Time-resolved changes in MRSA viability during phage infection were quantified using an agar-based CFU enumeration assay (Figure 2J). In the absence of phage, MRSA cultures maintained relatively stable CFU/mL values throughout the 2 h observation period, indicating sustained bacterial viability under the assay conditions. In phage-treated cultures, bacterial killing followed a time- and MOI-dependent pattern. After an initial increase in CFU/mL during the first 20–30 min post-infection, a marked reduction in viable bacteria was observed at approximately 40 min, indicating the onset of detectable phage-mediated killing. This decrease was most pronounced at higher MOIs, particularly MOI 10. Following this transient decline, CFU/mL partially rebounded and peaked again around 60 min, suggesting continued replication of surviving bacterial subpopulations. However, from 60 to 120 min post-infection, cultures infected at higher MOIs consistently maintained lower viable counts than those infected at lower MOIs or untreated controls. These data demonstrate active phage infection and sustained suppression of MRSA growth, with measurable lytic effects detectable as early as 40 min post-infection.

### 3.7. Compatibility of Bacteriophages with Organic Solvents and Surfactants

The stability of bacteriophages SA-W2 and MRSA-W3 in the presence of commonly used organic solvents and surfactants was evaluated using spot test-based lytic activity assays (Figure 2E–I). Both phages retained infectivity following exposure to sodium dodecyl sulfate (SDS) and Triton X-100, as evidenced by persistent clear or turbid lysis zones across tested dilutions. SA-W2 exhibited clear and consistent lysis after SDS and Triton X-100 exposure (Figure 2E,F), indicating strong tolerance to both anionic and non-ionic surfactants. Similarly, MRSA-W3 maintained plaque-forming activity following treatment with SDS and Triton X-100, although lysis patterns were occasionally more diffuse compared with SA-W2 (Figure 2G,H). In contrast, Tween 80 preserved detectable plaque formation for both phages without marked suppression of lytic activity, while dimethyl sulfoxide (DMSO) supported phage infectivity only at low to moderate concentrations. Ethanol exposure resulted in a pronounced reduction or loss of lytic activity, particularly at concentrations ≥ 10%, indicating limited phage stability under these conditions (Figure 2I). Based on the combined lytic stability profiles, surfactant compatibility, and formulation performance, SDS and Triton X-100 were ranked as the most suitable excipients for phage-based surface disinfection applications, followed by Tween 80 and DMSO, whereas ethanol showed limited suitability (Table 4).

### 3.8. Surface Disinfection Efficacy of Phage–Surfactant Formulations

**Figure 3:**
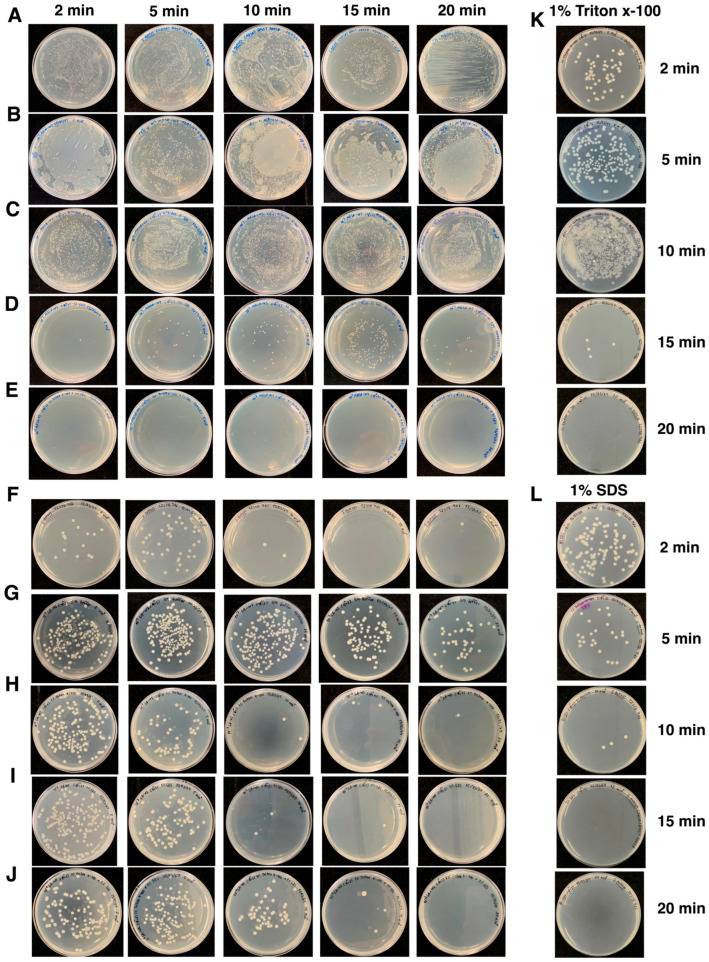
Surface disinfection efficacy of phage–surfactant formulations on glass-based container surfaces. *Staphylococcus aureus* strains were exposed to bacteriophages MRSA-W3 or SA-W2 (10^7^ PFU/mL), alone or in combination with surfactants, on container surfaces for 2, 5, 10, 15, and 20 min. Surviving bacteria were quantified as CFU/mL using the total plate count method. Panels show representative outcomes: (**A**) *S. aureus* (MRSA) DMST 20654 (control); (**B**) MRSA-W3 alone; (**C**) MRSA-W3 in 1% Triton X-100; (**D**) MRSA-W3 in 1% SDS; (**E**) MRSA-W3 in 1% Triton X-100 + 1% SDS; (**F**) *S. aureus* TISTR 746 (control); (**G**) SA-W2 alone; (**H**) SA-W2 in 1% Triton X-100; (**I**) SA-W2 in 1% SDS; (**J**) SA-W2 in 1% Triton X-100 + 1% SDS; (**K**) *S. aureus* TISTR 746 in 1% Triton X-100 (surfactant control); and (**L**) *S. aureus* TISTR 746 in 1% SDS (surfactant control).

Surface disinfection assays conducted on glass-based container surfaces demonstrated time-dependent reductions in recoverable *Staphylococcus aureus* following treatment with selected phage–surfactant formulations (Figure 3A–E,K,L; Table 5 and Table S5a,b). For MRSA-W3, combinations with 1% Triton X-100 or 1% SDS (applied at a 10^7^ PFU/mL) resulted in a rapid decrease in viable bacteria. Mean recoverable counts declined from 2.48 log_10_ CFU/cm^2^ at baseline to 0.30 log_10_ CFU/cm^2^ within 15 min, corresponding to a maximum Δlog_10_ reduction of 2.18 relative to untreated control surfaces. Log-reduction values were calculated only at time points where untreated controls yielded quantifiable CFU, in accordance with standard carrier-test practice. In contrast, SA-W2–based formulations showed greater variability and did not consistently outperform surfactant-only controls across time points (Figure 3F–J; Table 5 and Table S5b). In several conditions, untreated control surfaces reached the limit of detection at early time points, precluding valid Δlog_10_ calculations. For these cases, results are therefore reported as absolute CFU recovery rather than log-reduction values. Apparent negative Δlog_10_ values observed in preliminary calculations reflected this limitation rather than enhanced bacterial survival and were not interpreted as biologically meaningful effects. Across all conditions, variability among biological replicates is presented explicitly in Table 5 and Supplementary Table S5a,b to reflect experimental dispersion and avoid selective reporting. Overall, these results indicate a formulation-dependent enhancement of antibacterial activity for MRSA-W3 when combined with selected surfactants under this carrier-like surface model, while highlighting the importance of recovery limits and assay context when interpreting short-duration surface disinfection data. Surfactant-only controls (1% SDS or 1% Triton X-100) produced rapid reductions in recoverable MRSA, and at later time points frequently approached the limit of detection, consistent with their known bactericidal activity. The measurable added value of phage inclusion was therefore most evident at early exposure times (2–15 min), where phage–surfactant formulations, particularly MRSA-W3 combined with SDS or Triton X-100, achieved greater and more rapid reductions in viable counts compared with surfactant-only controls under conditions permitting valid Δlog_10_ calculation. At later time points where surfactant-only controls yielded zero or near-zero CFU recovery, Δlog_10_ reductions were not calculated, and results are reported as absolute CFU counts to avoid overinterpretation.

### 3.9. Genome Assembly and Taxonomic Profiling of MRSA-Infecting Phage MRSA-W3

**Figure 4:**
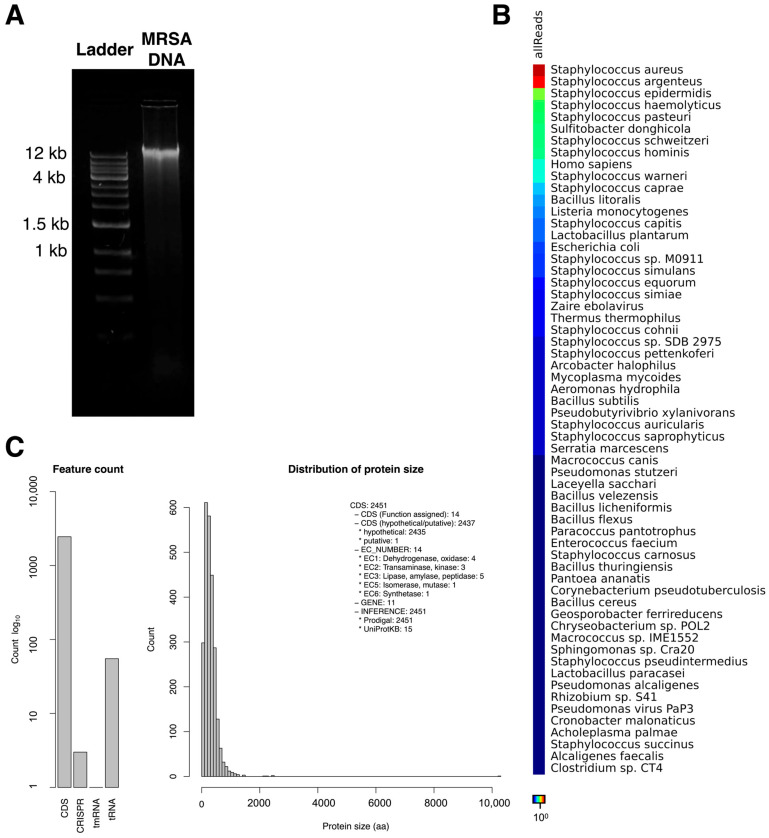
Molecular and infection-coupled sequencing-based characterization of phage-associated contigs derived from MRSA-W3–infected Staphylococcus aureus cells. (**A**) Agarose gel electrophoresis showing DNA banding patterns obtained from *Staphylococcus aureus* cells following infection with bacteriophage MRSA-W3. (**B**) Heat map illustrating bacterial taxonomic profiling based on sequencing read data. Relative abundance of bacterial taxa was determined by comparison with reference databases, with color intensity indicating abundance (red to orange, high abundance; yellow to green, moderate abundance). (**C**) De novo assembly yielded 39 contigs (≥1000 bp), with a largest contig size of 459,358 bp, a total assembled length of approximately 2.73 Mb, an N50 value of 186,171 bp, an L50 of 5, and a GC content of 32.25%.

An infection-coupled sequencing approach was used to explore the molecular and genomic features associated with bacteriophage MRSA-W3 following infection of *Staphylococcus aureus* (Figure 4). Agarose gel electrophoresis revealed additional DNA bands in infected samples compared with DNA marker (Figure 4A). Taxonomic profiling of sequencing reads showed that the majority of classified reads corresponded to *S. aureus*, reflecting the expected dominance of host genomic material in infection-based sequencing datasets (Figure 4B; Table 6). Across multiple complementary bioinformatic tools, including GOTTCHA, PANGIA, BWA, Kraken2, and Centrifuge, *S. aureus* strains such as JKD6159, MRSA252, and MSHR1132 were consistently identified as the most abundant bacterial references. These findings indicate effective capture of host DNA and provide context for interpreting phage-associated signals within the dataset. Despite the predominance of bacterial reads, a subset of viral sequences was consistently detected. Viral taxonomic assignment identified *Staphylococcus* bacteriophages as the principal viral components, with *Staphylococcus phage* StauST398-5 and *Staphylococcus phage* phi2958PVL recurrently detected across classification pipelines (Table 6). Species-level viral profiling further indicated that these two phages accounted for the majority of viral reads, while additional *Staphylococcus* phages were detected at lower relative abundances (Table 7). Low-frequency non-*Staphylococcus* viral assignments were observed but were considered background signals given their minimal read counts. De novo assembly of infection-coupled sequencing reads yielded a host-dominated metagenomic assembly, reflecting the abundance of bacterial DNA relative to phage-derived sequences under the applied extraction strategy (Figure 4C; Table S6). The assembly comprised 87 contigs, with a largest contig size of 459,358 bp, an N50 of 186,171 bp, and a total assembled length of approximately 2.74 Mb. Genome quality assessment indicated high completeness (99.99%) and low estimated contamination (0.13%), with a GC content of approximately 32% and a coding density of 0.84. These metrics support the technical robustness of the assembly, while acknowledging that the infection-coupled approach may include residual host-derived sequences. Accordingly, this dataset is hereafter referred to as an infection-coupled assembly, and phage-associated sequences are discussed at the contig level rather than as a complete phage genome. While the assembled genome shows similarity to previously described *Staphylococcus* phages, particularly PVL-associated and ST398-related lineages, further targeted phage-only sequencing and functional annotation would be required to fully resolve the taxonomic placement and genomic architecture of MRSA-W3. Nonetheless, the genomic data provide supportive molecular context for the phenotypic and functional activities observed in this study.

### 3.10. Prophage-like Region and Genomic Relatedness of the MRSA-Infecting Phage MRSA-W3

**Figure 5:**
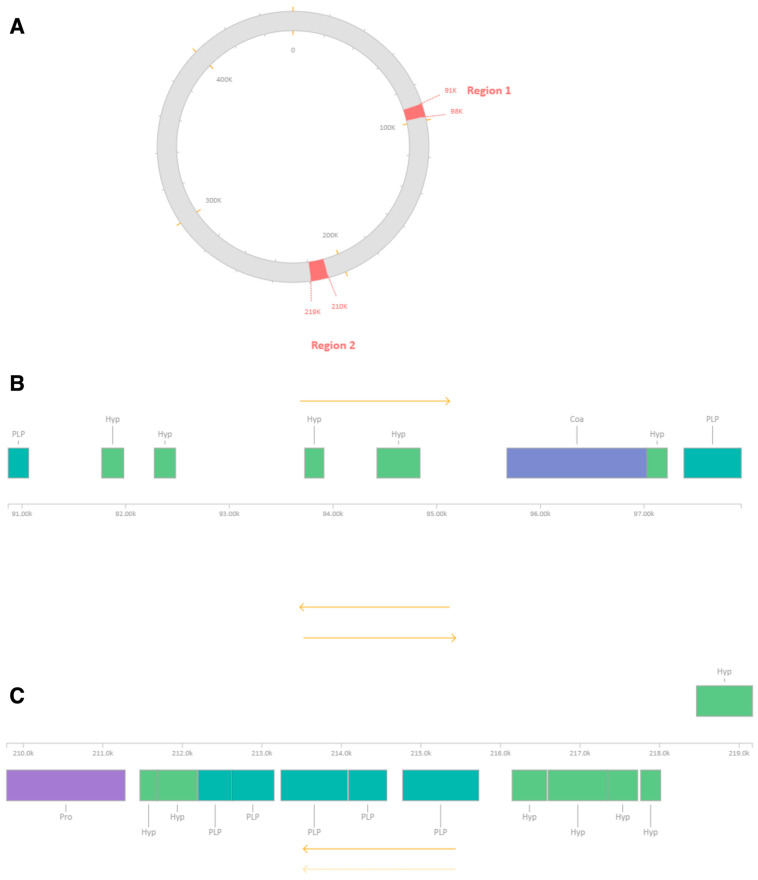

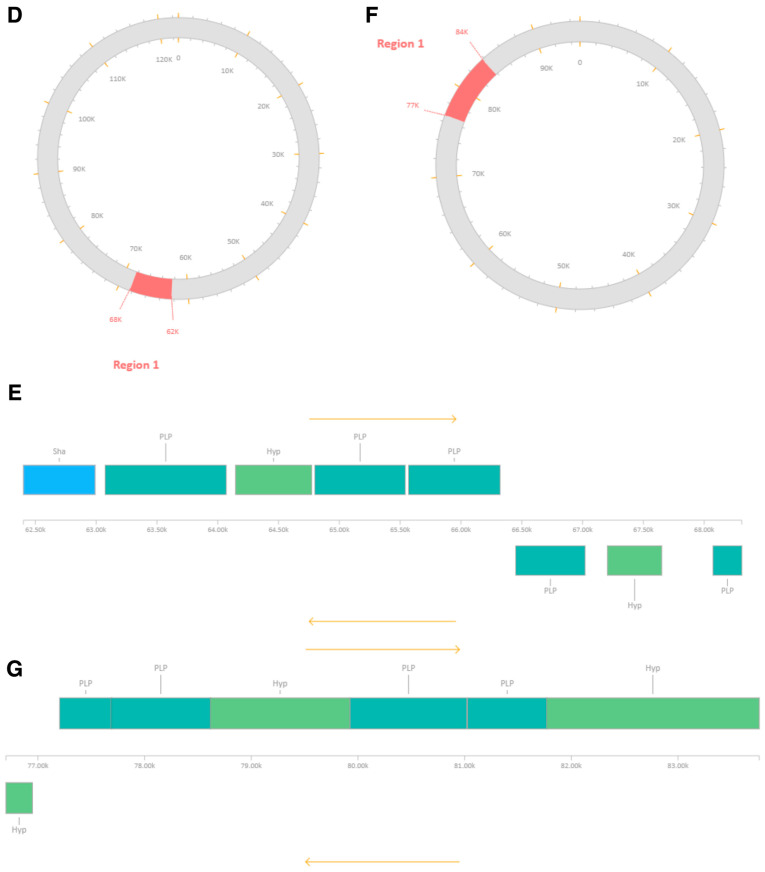
Prophage-like regions identification and genomic distribution within the MRSA-Infecting Phage MRSA-W3 genome. Phage analysis identified multiple prophage-like regions within assembled contigs integrated into the MRSA-Infecting Phage MRSA-W3 genome. Prophage-like region sequences were detected in three contigs, designated MRSA-W3_001, MRSA-W3_007, and MRSA-W3_009. At least one prophage region was identified in each contig, with MRSA-W3_001 harboring two distinct prophage-like regions within assembled contigs (**A**–**C**), whereas MRSA-W3_007 (**D**,**E**) and MRSA-W3_009 (**F**,**G**) each contained a single prophage-like region. The yellow arrow represents the direction of DNA.

**Figure 6:**
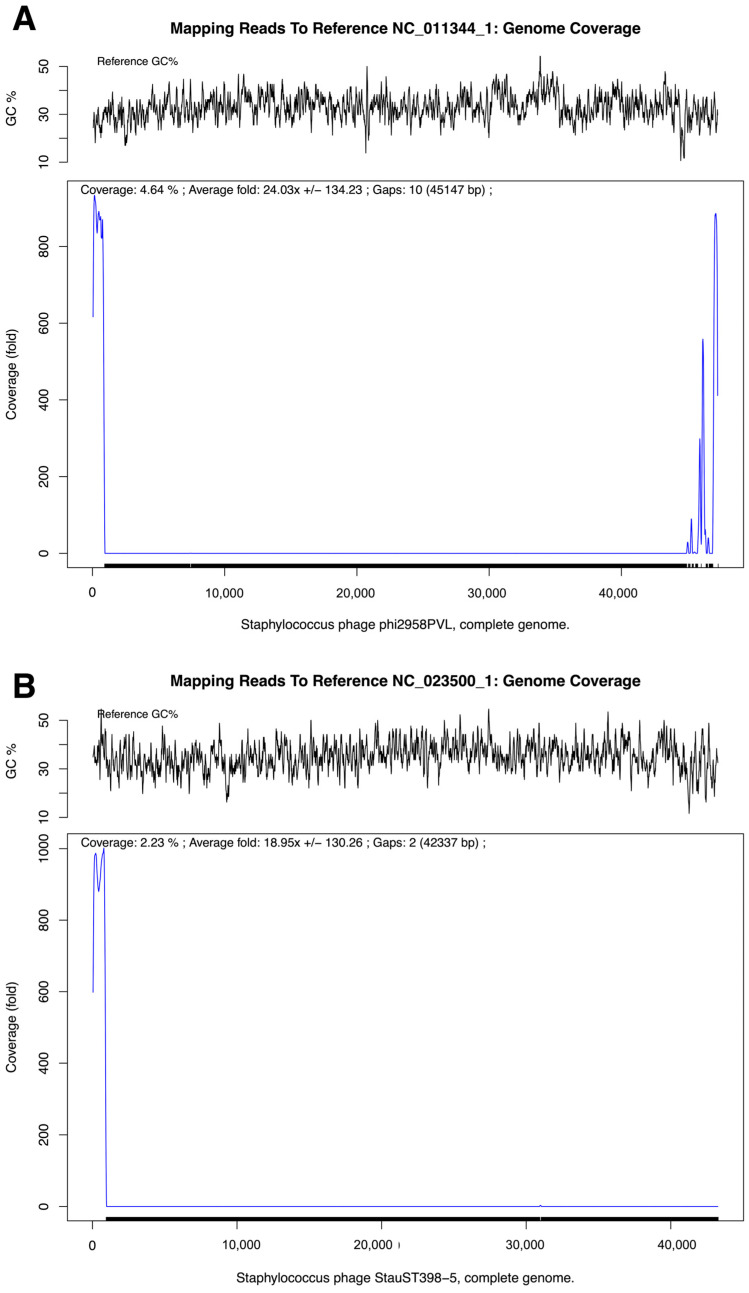
Mapping of MRSA-Infecting Phage MRSA-W3 sequencing reads to reference bacteriophage genomes. (**A**) Genome-wide read coverage and GC content profiles obtained by mapping MRSA-W3 sequencing reads to the reference genome of *Staphylococcus* phage phi2958PVL (NC_011344.1). (**B**) Genome-wide read coverage and GC content profiles obtained by mapping MRSA-W3 sequencing reads to the reference genome of *Staphylococcus* phage StauST398-5 (NC_023500.1).

Whole-genome sequencing and comparative analyses were performed to further characterize the genetic architecture of the MRSA-infecting phage MRSA-W3 and to assess its relationship to previously described staphylococcal phages. Prophage-like regions were identified within selected contigs based on sequence similarity and the presence of phage hallmark genes; however, these features were detected within a mixed host–phage assembly and are interpreted conservatively as remnants of mosaic phage ancestry rather than evidence of functional temperate behavior. Using prophage prediction tools, multiple prophage-like regions were identified within the assembled MRSA-W3 genome (Figure 5 and Table 8). These regions were distributed across three contigs—MRSA-W3_001, MRSA-W3_007, and MRSA-W3_009—each harboring one or more prophage elements. In contig MRSA-W3_001, two distinct prophage-like regions within assembled contigs were detected, spanning approximately 7.0 kb and 9.3 kb, respectively. Both regions were classified as incomplete prophages based on prophage scores and the absence of a full complement of essential phage genes. Nevertheless, these regions retained hallmark phage-associated genes, including capsid and scaffold proteins, integrase, terminase, and tail-associated components, suggesting remnants of historical phage integration events. Similarly, contigs MRSA-W3_007 and MRSA-W3_009 each contained a single incomplete prophage region (5.9 kb and 7.0 kb, respectively), characterized by a limited number of coding sequences and mosaic gene compositions (Table 8 and Tables S7–S9). Comparative annotation indicated that the closest related phages varied among prophage-like regions within assembled contigs, including *Staphylococcus* phages SA97, SPβ-like phages, phiSa119, and PaV-LD, reflecting the genetic heterogeneity and modular nature of phage genomes. All identified prophage-like regions within assembled contigs were classified as incomplete, supporting the interpretation that these elements represent residual or non-inducible prophage fragments rather than intact temperate phages. To further contextualize MRSA-W3 within known staphylococcal phage lineages, sequencing reads were mapped to reference bacteriophage genomes. Read-mapping analysis demonstrated partial but consistent coverage against *Staphylococcus* phage phi2958PVL and *Staphylococcus* phage StauST398-5 (Figure 6 and Table 9). Mapped regions exhibited moderate average coverage without detectable single-nucleotide variants or insertions/deletions, indicating sequence similarity rather than identity. These findings suggest that MRSA-W3 shares conserved genomic segments with PVL-associated and ST398-related phages, while maintaining a distinct overall genomic structure. Taxonomic profiling using multiple complementary bioinformatic tools further supported this interpretation (Table 6 and Table 7). Viral read assignments were dominated by *Staphylococcus* phages, particularly StauST398-5 and phi2958PVL, whereas low-abundance viral reads assigned to unrelated taxa were considered background signals. Genome quality assessment indicated high completeness (>99.9%) and low contamination (<0.2%), supporting the reliability of the assembled sequence (Table S6). Taken together, these genomic analyses indicate that MRSA-W3 represents a genetically distinct staphylococcal phage that contains multiple incomplete prophage-like regions and shares partial homology with known *Staphylococcus* phages, including PVL-associated and ST398-related lineages. While these data offer insight into the genomic composition and evolutionary context of MRSA-W3, they are insufficient to infer functional temperate behavior and are accordingly interpreted conservatively as reflecting mosaic phage ancestry rather than active prophage induction.

## 4. Discussion

This study evaluated environmentally derived *Staphylococcus*-infecting bacteriophages with emphasis on their applied potential for MRSA surface control, integrating phenotypic antimicrobial activity, formulation compatibility, and genomic context. Collectively, the findings indicate that selected phages, particularly MRSA-W3 and SA-W2, exhibit reproducible lytic activity against MRSA, measurable anti-biofilm effects, and formulation-dependent surface decontamination efficacy under controlled experimental conditions, while also delineating limitations relevant to translation into infection prevention practice.

Time-resolved infection assays revealed a rapid, multiplicity-of-infection-dependent reduction in viable MRSA, with the most pronounced decline occurring approximately 40 min after phage exposure. This temporal pattern is consistent with early stages of productive lytic infection reported for staphylococcal phages and supports the use of CFU-based kinetics to capture phage-mediated killing before equilibrium between bacterial regrowth and phage replication is reached [15]. The partial rebound in bacterial counts observed at later time points, particularly at lower MOIs, likely reflects survival of subpopulations and dynamic phage–host interactions rather than experimental failure, a phenomenon widely reported in short-term in vitro phage studies [64].

Beyond planktonic cultures, both MRSA-W3 and SA-W2 produced statistically significant but heterogeneous reductions in biofilm biomass across clinical isolates. Such inter-strain variability is expected, given the diversity of biofilm regulatory pathways, matrix composition, and surface adherence phenotypes in *S. aureus* [65]. Importantly, median inhibition values remained consistently greater than zero, indicating a reproducible anti-biofilm signal even in the absence of complete biofilm eradication. These results align with recent studies demonstrating that bacteriophages can interfere with early biofilm formation or reduce biofilm mass, while mature biofilms often require combined or formulation-based approaches for more extensive disruption [20,66,67]. Because crystal violet staining quantifies total biomass, it does not differentiate between viable and non-viable cells or resolve phage replication kinetics within biofilms.

Phage compatibility with formulation components represents a critical translational consideration. In this study, both phages retained infectivity following exposure to SDS, Triton X-100, and Tween 80, whereas ethanol and higher concentrations of organic solvents caused rapid loss of activity. This pattern is consistent with recent reports showing that non-ionic and anionic surfactants may preserve phage capsid integrity, while alcohol-based disinfectants disrupt virion structure and nucleic acids [31,68,69]. In surface disinfection assays, phage–surfactant formulations produced statistically significant reductions in recoverable MRSA, achieving Δlog_10_ reductions comparable to those reported for emerging non-antibiotic antimicrobial adjuncts, although remaining below the thresholds typically required for stand-alone chemical disinfectants [70]. Therefore, observed Δlog_10_ reductions should be interpreted as adjunct antimicrobial effects rather than evidence of disinfectant equivalence. While formal neutralization is required in EN/ASTM carrier tests, the present approach prioritizes comparative internal controls to support proof-of-concept evaluation. The surface model further indicates that the primary contribution of bacteriophage addition lies in enhancing early killing kinetics rather than replacing surfactant-driven disinfection. While surfactants alone achieved substantial reductions at later time points, inclusion of MRSA-W3 accelerated bacterial reduction during the initial exposure period, supporting its role as an adjunct rather than a standalone disinfectant. Accordingly, phage–surfactant formulations are interpreted as providing context-dependent, adjunct-level benefit, and not as equivalents to standardized chemical disinfectants under EN or ASTM performance criteria.

Whole-genome sequencing of MRSA-W3 provided additional context for these phenotypic observations [34,35,36,37]. Genome-wide analyses consistently support the classification of MRSA-W3 as a *Staphylococcus*-infecting bacteriophage, with no identifiable genomic features indicative of adaptation to Gram-negative hosts. This genomic assignment is concordant with the phenotypic data, which demonstrate productive infection only within *Staphylococcus* species. The high completeness and low contamination of the assembly support the robustness of the infection-coupled sequencing strategy. Taxonomic profiling consistently linked MRSA-W3 to known staphylococcal phage lineages, while the detection of incomplete prophage-like regions and mosaic genomic features likely reflects historical recombination and shared evolutionary ancestry rather than functional temperate behavior. Similar genomic architectures have been described in environmentally isolated phages that remain functionally lytic, underscoring the need for cautious interpretation of prophage annotations in applied phage research [71,72,73,74]. While the infection-coupled sequencing approach yielded a high-quality assembly with near-complete coverage, host DNA dominance limits resolution, and virion-enriched sequencing will be required for definitive MRSA-W3 genome closure. Because bacterial genomic DNA substantially exceeded virion-derived DNA in the infection-coupled extracts, definitive closure of the MRSA-W3 genome will require virion-enriched sequencing strategies. Putative prophage-like regions detected within the infection-coupled assembly were interpreted conservatively as remnant or mosaic phage-derived sequences embedded in host DNA, rather than as evidence of functional lysogeny or temperate phage behavior. Virion-enriched sequencing and host DNA depletion will be required in future studies to achieve definitive genome closure and higher-confidence structural annotation.

Although Δlog_10_ reductions up to 2.18 CFU/cm^2^ were observed under optimized conditions, these values do not meet the regulatory thresholds for standalone surface disinfectants, but instead support the potential role of phage–surfactant formulations as adjunct or complementary control measures. From an infection prevention standpoint, these findings support the concept that bacteriophages may serve as targeted, non-antibiotic adjuncts for MRSA surface control, particularly in settings where biofilms and disinfectant tolerance contribute to environmental persistence. At the same time, the results highlight the importance of standardized efficacy testing, longer-term stability assessments, and evaluation under realistic surface and organic load conditions before broader implementation, consistent with current guidance and emerging regulatory discussions surrounding phage-based antimicrobial applications [13,75,76,77,78].

## 5. Conclusions

This study demonstrates that environmentally derived *Staphylococcus*-infecting bacteriophages MRSA-W3 and SA-W2 exhibit reproducible lytic activity against MRSA, measurable inhibition of biofilm formation, and statistically significant reductions in recoverable surface-associated bacteria under controlled experimental conditions. Phage-mediated killing was rapid and MOI-dependent, and compatible surfactant formulations supported consistent Δlog_10_ reductions indicative of adjunct-level antimicrobial performance, rather than stand-alone disinfectant equivalence. Phage-related added value over surfactant-only controls was observed primarily at early exposure times (2–15 min), whereas reductions at later time points were largely attributable to surfactant activity and were therefore reported without Δlog_10_ comparison. Phage stability in the presence of selected anionic and non-ionic surfactants supports formulation feasibility, while whole-genome analysis of MRSA-W3 revealed a high-quality mosaic genome with incomplete prophage-like elements that were interpreted conservatively as reflecting shared evolutionary ancestry rather than functional temperate behavior. Collectively, these findings support the further development of bacteriophage-based formulations as non-antibiotic adjuncts for MRSA surface control, with future studies required to align efficacy testing to standardized EN/ASTM frameworks, assess long-term stability and resistance dynamics, and define regulatory pathways for infection-prevention use.

## Figures and Tables

**Table 1:** Sampling locations and bacteriophage detection by spot test assay against *Staphylococcus* host strains.

Sample ID	Sample Type	Sampling Location	Geographic Coordinates (Lat, Long)	*S. epidermidis* TISTR 518	MRSA *S. aureus* DMST 20654	*S. aureus* TISTR 746
S1	Soil	Cattle shed, Lam Chi area	16.220521, 103.274104	+	+	+
S2	Soil	Rear area of cattle shed, Lam Chi	16.220521, 103.274104	+	+	+
S3	Soil	Cattle shed, village area	16.248027, 103.257684	−	+	+
S4	Soil	Pond side near cattle shed, village	16.248027, 103.257684	−	+	+
S5	Soil	Pond side, front of Mahasarakham University	16.247221, 103.256828	−	+	+
W1	Water	Water in front of cattle shed, Lam Chi	16.220521, 103.274104	−	+	+
W2	Water	Water behind cattle shed, Lam Chi	16.220521, 103.274104	+	+	+
W3	Water	Water within cattle shed, village	16.248027, 103.257684	−	+	+
W4	Water	Pond water near cattle shed, village	16.248027, 103.257684	−	+	+
W5	Water	Wastewater from public park, Mahasarakham University	16.247221, 103.256828	−	+	+

Notes: (+) Plaques observed in spot test assay; (−) no plaques observed.

**Table 2:** Host range of bacteriophages against clinical *S. aureus* isolates *(n* = 50) and cross-species activity to non-*Staphylococcus* strains (*n* = 7).

Phage Isolate	Susceptible/Tested	Host Range (%)	95% CI (Wilson)
Clinical *S. aureus* isolates (*n* = 50)			
MRSA-S1	20/50	40.0	27.6–53.8
MRSA-S2	25/50	50.0	36.8–63.2
MRSA-W3	39/50	78.0	64.8–87.3
SA-S1	14/50	28.0	17.2–41.6
SA-S2	24/50	48.0	35.0–61.3
SA-W1	22/50	44.0	31.4–57.4
SA-W2	29/50	58.0	44.2–70.7
SA-W5	18/50	36.0	24.3–49.6
SE-W2	17/50	34.0	22.5–47.5
Non-*Staphylococcus* strains (*n* = 7)			
MRSA-W3	5/7	71.4	35.9–91.8
SA-W2	5/7	71.4	35.9–91.8
Other phages	0/7	0.0	0.0–35.4

Note: Binomial 95% confidence intervals (Wilson method).

**Table 3:** Summary of thermal stability classification of bacteriophages MRSA-W3 and SA-W2.

Bacteriophage	Temperature Range (°C)	Stability Classification	Evidence from Plaque Assay
MRSA-W3	25	Stable	Plaques detected at all exposure times with countable plaques up to 10^4^ PFU/mL
	37	Stable	Confluent plaques observed at all dilutions and time points
	50	Stable	Plaques consistently detected across all dilutions and exposure times
	60	Stable	No loss of plaque-forming ability at any dilution or time point
SA-W2	25	Stable	Plaques observed at all phage concentrations (10^9^–10^4^ PFU/mL) and exposure times
	37	Partially stable	Loss of plaques at 10^4^ PFU/mL across all time points
	50	Stable	Plaques retained at all dilutions and exposure times
	60	Stable	Detectable plaques maintained, including high dilutions at prolonged exposure

Note: Criteria used for classification. Stable: Plaques detected at all phage concentrations (10^9^–10^4^ PFU/mL) across all exposure times. Partially stable: Plaques lost only at 10^4^ PFU/mL but retained at higher concentrations. Unstable: No plaques detected at ≥2 consecutive dilutions or complete loss of lytic activity.

**Table 4:** Formulation suitability ranking for surface disinfection applications.

Rank	Solvent/Surfactant	Overall Suitability	Scientific Rationale
1	SDS	Highly suitable	Consistent clear lysis (C) indicates maximal lytic activity; ideal for surface disinfection where rapid killing is desired
2	Triton X-100	Highly suitable	Strong clear lysis for SA-W2 and stable plaques for MRSA-W3; effective non-ionic surfactant
3	Tween 80	Suitable	Maintains plaque-forming activity without suppressing infectivity; formulation-friendly
4	DMSO	Conditionally suitable	Supports activity at low–moderate concentrations; not ideal at high levels
5	Ethanol (EtOH)	Limited suitability	Rapid loss of activity at ≥10% limits disinfectant applications

**Table 5:** Time-dependent log_10_ reduction in viable *Staphylococcus aureus* following treatment with phage–surfactant formulations on glass-based container surfaces.

Phage/Strain	Treatment	Time (min)	CFU	log_10_ CFU	Δlog_10_ Reduction vs. Control
MRSA-W3	Control (no treatment)	2	300	2.48	–
		5	300	2.48	–
		10	300	2.48	–
		15	300	2.48	–
		20	248	2.40	–
MRSA-W3	MRSA-W3 + 1% Triton X-100 + 1% SDS (10^−3^)	2	47	1.67	0.81
		5	12	1.08	1.40
		10	15	1.18	1.30
		15	2	0.30	2.18
		20	3	0.48	1.92
SA-W2	Control (no treatment)	2	16	1.20	–
		5	41	1.61	–
		10	5	0.70	–
		15	0 *	0.00	–
		20	2	0.30	–
SA-W2	SA-W2 + 1% Triton X-100 + 1% SDS (10^−3^)	2	71	1.85	−0.65
		5	210	2.32	−0.71
		10	7	0.85	−0.15
		15	8	0.90	−0.90
		20	3	0.48	−0.18

Note: * For statistical handling and plotting, zero CFU values were treated as the detection limit. Δlog_10_ reductions were calculated relative to time-matched untreated controls.

**Table 6:** Taxonomic profiling results of MRSA-infected phage MRSA-W3 genome using multiple bioinformatic tools.

Tool	Database/Algorithm	Reads	% Reads	Taxonomic Level	Top 1 Hit	Top 2 Hit	Top 3 Hit	Top 4 Hit	Top 5 Hit
GOTTCHA (Bacteria)	gottcha-strDB-b	547,027	63.5	Strain	*S. aureus* MSHR1132	*S. aureus* MRSA252	*S. warneri* SG1	*S. aureus* CN1	*S. aureus* JKD6159
GOTTCHA (Virus)	gottcha-strDB-v	762	0.1	Strain	*Staphylococcus* phage StauST398-5	*Staphylococcus* phage phi2958PVL	*Staphylococcus* phage phiPVL-CN125	*Staphylococcus* phage PT1028	*Staphylococcus* phage 42e
PANGIA	k-mer + alignment	264,927	30.8	Strain	*S. aureus* JKD6159	*S. aureus* M1169	*S. aureus* NCTC8325	*S. aureus* NCTC8726	*S. aureus* NCTC5663
MetaPhlAn4	Marker gene-based	0	0.0	Strain	N/A	N/A	N/A	N/A	N/A
BWA	Read mapping	130,864	15.2	Strain	*S. aureus* JKD6159	*S. aureus* MSSA476	*S. aureus* MRSA252	*S. aureus* USA300	*S. aureus* ST398
Kraken2	k-mer classification	11,344	1.3	Strain	*S. aureus* JKD6159	Unclassified *Staphylococcus*	*S. aureus* TCH60	*S. aureus* ST398	*S. epidermidis* PM221
Centrifuge	Index-based classification	1701	0.2	Strain	*S. aureus* JKD6159	*S. epidermidis* PM221	*S. aureus* T0131	*S. aureus* ST398	*S. aureus* JKD6159
DIAMOND	Protein alignment	0	0.0	Strain	N/A	N/A	N/A	N/A	N/A

Note: Taxonomic classification was performed at the strain level using multiple complementary bioinformatic tools. Read counts and relative abundance (% reads) represent the proportion of sequencing reads assigned by each tool. “N/A” indicates that no taxonomic assignment was obtained.

**Table 7:** Read-level viral taxonomic profiling of sequencing reads associated with the MRSA-Infecting Phage MRSA-W3 genome.

Taxonomic Level	Viral Species	Read Count	Relative Abundance (%)	Plasmid-Associated Reads (%)
Species	*Staphylococcus* phage StauST398-5	274	59.1	0.0
Species	*Staphylococcus* phage phi2958PVL	150	30.3	0.0
Species	*Staphylococcus* phage phiPVL-CN125	52	7.4	0.0
Species	*Staphylococcus* phage PT1028	233	1.7	0.0
Species	*Staphylococcus* phage 42e	77	1.4	0.0
Species	Zaire ebolavirus	3	0.1	0.0

Note: Viral taxonomic assignment was performed at the species level based on sequence similarity to reference databases. Relative abundance represents the proportion of viral reads assigned to each taxon. Low-abundance non-*Staphylococcus* viral reads were considered background signals.

**Table 8:** Comparative summary of prophage-like regions within assembled contigs identified in the MRSA-Infecting Phage MRSA-W3 genome.

Contig	Prophage Like Region	Genomic Position (bp)	Region Length (kb)	Completeness	Prophage Score	No. of CDSs	Closest Related Phage	GC Content (%)	Key Genetic Features
MRSA-W3_001	Region 1	90,867–97,938	~7.0	Incomplete	20	8	*Staphylococcus* phage SA97 (NC_029010)	27.23	Capsid proteins, scaffold protein, DNA-binding protein
MRSA-W3_001	Region 2	209,791–219,168	~9.3	Incomplete	20	13	*Staphylococcus* phage SPβ-like (NC_029119)	31.69	Integrase, terminase, tail-associated proteins, nucleotide metabolism enzymes
MRSA-W3_007	Region 1	62,402–68,309	~5.9	Incomplete	20	8	*Staphylococcus* phage phiSa119 (NC_025460)	29.77	Structural proteins, holin, enterotoxin/exotoxin-related genes
MRSA-W3_009	Region 1	76,700–83,762	~7.0	Incomplete	10	7	Planktophage PaV-LD (NC_016564)	33.16	Metabolic/regulatory proteins; mosaic phage signatures

Notes: Prophage-like regions within assembled contigs were predicted based on sequence similarity and the presence of phage hallmark genes. All regions were classified as incomplete prophages, indicating remnant or non-inducible phage elements. CDS: coding sequence.

**Table 9:** Mapping of MRSA-Infecting Phage MRSA-W3 Sequencing Reads to Reference Bacteriophage Genomes.

Reference Phage	Accession No.	Genome Length (bp)	GC Content (%)	Mapped Reads (n)	Reads (%)	Average Coverage (×)	Gaps (n)	SNVs	INDELs
*Staphylococcus* phage phi2958PVL	NC_011344.1	47,342	33.04	9591	0.04	24.03	10	0	0
*Staphylococcus* phage StauST398-5	NC_023500.1	43,301	35.14	5598	0.02	18.95	2	0	0

Note: No single-nucleotide variants (SNVs) or insertions/deletions (INDELs) were detected in the mapped regions for either reference genome.

## Data Availability

All data supporting the findings of this study are available within the article and its Supplementary Materials.

## Supplementary Materials

The following supporting information can be downloaded at https://www.mdpi.com/article/10.3390/antibiotics15020155/s1. Table S1. Biochemical characteristics and antimicrobial susceptibility profiles of bacterial isolates used in this study. Table S2. Host range activity of isolated bacteriophages against *Staphylococcus aureus* strains and other bacterial species. Table S3. Anti-biofilm activity of bacteriophages SA-W2 and MRSA-W3 against clinical and reference bacterial isolates. Table S4a. Effect of temperature and exposure time on the stability of bacteriophage MRSA-W3. Table S4b. Effect of temperature and exposure time on the stability of bacteriophage SA-W2. Table S5a. Glass-based container surfaces disinfection efficacy of bacteriophage MRSA-W3 in organic solvent formulations. Table S5b. Surface disinfection efficacy of bacteriophage SA-W2 in organic solvent formulations. Table S6. Genome quality assessment and assembly statistics of *Staphylococcus aureus*-Infecting Phage MRSA-W3. Table S7a. Integrated summary of prophage-like regions within assembled contigs identified in contig MRSA-W3_001. Table S7b. Predicted coding sequences (CDSs) within prophage-like regions within assembled contigs 1 and 2 of contig MRSA-W3_001. Table S8a. Summary of prophage-like regions within assembled contigs identified in contig MRSA-W3_007. Table S8b. Predicted coding sequences (CDSs) within prophage-like regions within assembled contigs 1 of contig MRSA-W3_007. Table S9a. Summary of prophage-like regions within assembled contigs identified in contig MRSA-W3_009. Table S9b. Predicted coding sequences (CDSs) within prophage-like regions within assembled contigs 1 of contig MRSA-W3_009.
