# Supplementary material for: Bacteriophage-Based Control of Methicillin-Resistant Staphylococcus aureus: Anti-Biofilm Activity, Surface-Active Formulation Compatibility, and Genomic Context
Source: Antibiotics (Basel). 2026 Feb 2;15(2):155. doi: 10.3390/antibiotics15020155 (PMC12937358; doi:10.3390/antibiotics15020155)
Supplement: Supplementary file 1 [file antibiotics-15-00155-s001.zip › antibiotics-4092346-supplementary.pdf]

**Supplementary data**

**Table S1.** Biochemical characteristics and antimicrobial susceptibility profiles of bacterial isolates used in this study

| No. | Bacterial isolate | Source | Mannitol fermentation | Catalase | Oxidase | DNase | CX (30 µg) | COT (25 µg) | GEN (10 µg) | CD (10 µg) | E (10 µg) |
|---|---|---|---|---|---|---|---|---|---|---|---|
| 1 | *S. aureus* (MRSA) DMST 20654 | Reference strain | + | + | - | + | 19 (R) | 18 (S) | 25 (S) | 22 (S) | 17 (R) |
| 2 | *S. aureus* TISTR 746 | Reference strain | + | + | - | + | 25 (S) | 18 (S) | 25 (S) | 26 (S) | 18 (S) |
| 3 | *S. epidermidis* TISTR 518 | Reference strain | - | + | - | - | 27 (S) | 30 (S) | 25 (S) | 16 (S) | 15 (R) |
| 4 | *S. aureus* 56152 | Blood | + | + | - | + | 12 (R) | 28 (S) | 22 (S) | 14 (R) | 13 (R) |
| 5 | *S. aureus* 58036 | Blood | + | + | - | + | 13 (R) | 27 (S) | 21 (S) | 11 (R) | 12 (R) |
| 6 | *S. aureus* 58775 | Blood | + | + | - | + | 13 (R) | 23 (S) | 20 (S) | 7 (R) | 8 (R) |
| 7 | *S. aureus* 57563 | Blood | + | + | - | + | 22 (R) | 9 (R) | 9 (R) | 14 (R) | 8 (R) |
| 8 | *S. aureus* 57711 | Blood | + | + | - | + | 14 (R) | 20 (S) | 11 (R) | 9 (R) | 10 (R) |
| 9 | *S. aureus* 57263 | Blood | + | + | - | + | 10 (R) | 26 (S) | 23 (S) | 24 (S) | 24 (S) |
| 10 | *S. aureus* 57521 | Blood | + | + | - | + | 9 (R) | 24 (S) | 16 (S) | 9 (R) | 9 (R) |
| 11 | *S. aureus* 59031 | Blood | + | + | - | + | 8 (R) | 25 (S) | 22 (S) | 10 (R) | 10 (R) |
| 12 | *S. aureus* 59762 | Blood | + | + | - | + | 20 (R) | 9 (R) | 10 (R) | 12 (R) | 6 (R) |

| | | | | | | | | | | | |
|---|---|---|---|---|---|---|---|---|---|---|---|
| 13 | *S. aureus* 58737 | Blood | + | + | - | + | 10 (R) | 32 (S) | 23 (S) | 7 (R) | 13 (R) |
| 14 | *S. aureus* 58747 | Blood | + | + | - | + | 22 (R) | 23 (S) | 8 (R) | 9 (R) | 8 (R) |
| 15 | *S. aureus* 58506 | CSF | + | + | - | + | 16 (R) | 25 (S) | 23 (S) | 10 (R) | 10 (R) |
| 16 | *S. aureus* 58736 | Blood | + | + | - | + | 14 (R) | 22 (S) | 21 (S) | 14 (R) | 8 (R) |
| 17 | *S. aureus* 57906 | Blood | + | + | - | + | 10 (R) | 20 (S) | 12 (R) | 12 (R) | 13 (R) |
| 18 | *S. aureus* 57030 | Blood | + | + | - | + | 15 (R) | 26 (S) | 11 (R) | 6 (R) | 8 (R) |
| 19 | *S. aureus* 57235 | Blood | + | + | - | + | 14 (R) | 8 (R) | 9 (R) | 7 (R) | 11 (R) |
| 20 | *S. aureus* 56624 | Blood | + | + | - | + | 12 (R) | 22 (S) | 22 (S) | 9 (R) | 13 (R) |
| 21 | *S. aureus* 58037 | Blood | + | + | - | + | 18 (R) | 9 (R) | 12 (R) | 7 (R) | 7 (R) |
| 22 | *S. aureus* 54760 | Blood | + | + | - | + | 16 (R) | 27 (S) | 20 (S) | 9 (R) | 10 (R) |
| 23 | *S. aureus* 54784 | Blood | + | + | - | + | 9 (R) | 20 (S) | 24 (S) | 12 (R) | 12 (R) |
| 24 | *S. aureus* 58063 | Blood | + | + | - | + | 8 (R) | 28 (S) | 21 (S) | 7 (R) | 9 (R) |
| 25 | *S. aureus* 58505 | Blood | + | + | - | + | 18 (R) | 20 (S) | 24 (S) | 8 (R) | 10 (R) |
| 26 | *S. aureus* 55446 | Blood | + | + | - | + | 14 (R) | 28 (S) | 24 (S) | 8 (R) | 11 (R) |
| 27 | *S. aureus* 56488 | Blood | + | + | - | + | 10 (R) | 9 (R) | 23 (S) | 12 (R) | 12 (R) |
| 28 | *S. aureus* 56400 | Blood | + | + | - | + | 9 (R) | 29 (S) | 20 (S) | 8 (R) | 8 (R) |

| | | | | | | | | | | | |
|---|---|---|---|---|---|---|---|---|---|---|---|
| 29 | *S. aureus* 56142 | Blood | + | + | - | + | 10 (R) | 24 (S) | 21 (S) | 10 (R) | 9 (R) |
| 30 | *S. aureus* 56266 | Blood | + | + | - | + | 22 (R) | 7 (R) | 10 (R) | 5 (R) | 10 (R) |
| 31 | *S. aureus* 56223 | Blood | + | + | - | + | 16 (R) | 22 (S) | 25 (S) | 11 (R) | 9 (R) |
| 32 | *S. aureus* 56686 | Blood | + | + | - | + | 14 (R) | 22 (S) | 10 (R) | 10 (R) | 12 (R) |
| 33 | *S. aureus* 57259 | Blood | + | + | - | + | 7 (R) | 29 (S) | 10 (R) | 9 (R) | 8 (R) |
| 34 | *S. aureus* A1244/19 | Blood | + | + | - | + | 15 (R) | 30 (S) | 21 (S) | 6 (R) | 6 (R) |
| 35 | *S. aureus* A855/19 | Sputum | + | + | - | + | 17 (R) | 31 (S) | 26 (S) | 6 (R) | 6 (R) |
| 36 | *S. aureus* A619/19 | Blood | + | + | - | + | 6 (R) | 30 (S) | 11 (R) | 6 (R) | 6 (R) |
| 37 | *S. aureus* A1257/19 | Blood | + | + | - | + | 12 (R) | 26 (S) | 20 (S) | 6 (R) | 6 (R) |
| 38 | *S. aureus* A1349/19 | Blood | + | + | - | + | 17 (R) | 30 (S) | 25 (S) | 6 (R) | 6 (R) |
| 39 | *S. aureus* A193/19 | Sputum | + | + | - | + | 6 (R) | 31 (S) | 10 (S) | 6 (R) | 6 (R) |
| 40 | *S. aureus* A192/19 | Urine | + | + | - | + | 16 (R) | 31 (S) | 24 (S) | 6 (R) | 6 (R) |
| 41 | *S. aureus* A21/19 61721 | Blood | + | + | - | + | 10 (R) | 25 (S) | 22 (S) | 6 (R) | 6 (R) |
| 42 | *S. aureus* A22/19 61722 | Pus | + | + | - | + | 16 (R) | 28 (S) | 23 (S) | 6 (R) | 6 (R) |
| 43 | *S. aureus* A36/19 61734 | Pus | + | + | - | + | 15 (R) | 30 (S) | 23 (S) | 6 (R) | 6 (R) |
| 44 | *S. aureus* A513/19 | Wound | + | + | - | + | 14 (R) | 28 (S) | 21 (S) | 6 (R) | 6 (R) |

| | | | | | | | | | | | |
|---|---|---|---|---|---|---|---|---|---|---|---|
| 45 | *S. aureus* A1277/19 | Tissue | + | + | - | + | 15 (R) | 28 (S) | 23 (S) | 6 (R) | 6 (R) |
| 46 | *S. aureus* A1276/19 | Pus | + | + | - | + | 6 (R) | 20 (S) | 6 (R) | 6 (R) | 6 (R) |
| 47 | *S. aureus* A1322/19 | Pus | + | + | - | + | 13 (R) | 28 (S) | 22 (S) | 6 (R) | 6 (R) |
| 48 | *S. aureus* A53/19 61747 | Pus | + | + | - | + | 15 (R) | 29 (S) | 24 (S) | 6 (R) | 6 (R) |
| 49 | *S. aureus* A1348/19 | Sputum | + | + | - | + | 12 (R) | 6 (R) | 15 (S) | 12 (R) | 25 (S) |
| 50 | *S. aureus* A618/19 | Blood | + | + | - | + | 15 (R) | 30 (S) | 25 (S) | 6 (R) | 6 (R) |
| 51 | *S. aureus* 56555 | Blood | + | + | - | + | 13 (R) | 20 (S) | 24 (S) | 30 (S) | 24 (S) |
| 52 | *S. aureus* 56568 | Blood | + | + | - | + | 31 (S) | 6 (R) | 23 (S) | 6 (R) | 6 (R) |
| 53 | *S. aureus* 56618 | Blood | + | + | - | + | 6 (R) | 6 (R) | 6 (R) | 6 (R) | 6 (R) |

**Note:** CX: Cefoxitin (30 µg): S ≥ 25, R ≤ 24; COT: Co-trimoxazole (25 µg): S ≥ 16, R ≤ 10; GEN: Gentamicin (10 µg): S ≥ 15, R ≤ 12; CD: Clindamycin (10 µg): S ≥ 21, R ≤ 14; E: Erythromycin (10 µg): S ≥ 23, R ≤ 13; Interpretation criteria: Susceptibility interpretations (S, R) were assigned according to CLSI guidelines based on inhibition zone diameters (mm).

**Table S2.** Host range activity of isolated bacteriophages against *Staphylococcus aureus* strains and other bacterial species.

| No. | Bacterial strain | Source | MRSA-S1 | MRSA-S2 | MRSA-W3 | SA-S1 | SA-S2 | SA-W1 | SA-W2 | SA-W5 | SE-W2 |
|---|---|---|---|---|---|---|---|---|---|---|---|
| 1 | *S. aureus* 56152 | Blood | + | + | + | − | + | + | + | − | + |
| 2 | *S. aureus* 58036 | Blood | + | + | + | − | + | + | + | + | − |
| 3 | *S. aureus* 58775 | Blood | + | + | + | − | + | + | + | + | + |
| 4 | *S. aureus* 57563 | Blood | − | + | + | − | + | + | + | + | + |
| 5 | *S. aureus* 57711 | Blood | − | + | + | − | + | + | + | + | + |
| 6 | *S. aureus* 57263 | Blood | + | + | + | − | + | − | + | + | − |
| 7 | *S. aureus* 57521 | Blood | + | + | + | − | + | + | + | + | + |
| 8 | *S. aureus* 59031 | Blood | + | + | + | − | + | + | + | + | + |
| 9 | *S. aureus* 59762 | Blood | + | + | + | − | + | + | + | + | + |
| 10 | *S. aureus* 58737 | Blood | − | + | + | − | + | + | + | − | − |
| 11 | *S. aureus* 58747 | Blood | + | − | − | − | − | + | − | − | + |
| 12 | *S. aureus* 58506 | CSF | + | − | − | − | + | + | − | − | + |
| 13 | *S. aureus* 58736 | Blood | + | + | + | + | + | + | + | + | + |
| 14 | *S. aureus* 57906 | Blood | − | + | + | + | − | − | + | + | − |
| 15 | *S. aureus* 57030 | Blood | − | + | + | + | + | + | + | + | + |
| 16 | *S. aureus* 57235 | Blood | + | + | + | + | + | + | + | + | + |
| 17 | *S. aureus* 56624 | Blood | − | − | − | − | − | − | − | − | − |
| 18 | *S. aureus* 58037 | Blood | + | + | + | + | + | + | + | + | − |
| 19 | *S. aureus* 54760 | Blood | − | − | + | + | − | + | + | + | − |
| 20 | *S. aureus* 54784 | Blood | + | + | + | + | + | + | + | + | + |
| 21 | *S. aureus* 58063 | Blood | + | + | + | − | + | + | − | − | − |

| | | | | | | | | | | | |
|---|---|---|---|---|---|---|---|---|---|---|---|
| 22 | *S. aureus* 58505 | Blood | + | + | + | + | + | + | + | + | − |
| 23 | *S. aureus* 55446 | Blood | + | + | + | − | + | + | + | + | + |
| 24 | *S. aureus* 56488 | Blood | + | + | + | − | + | + | + | − | + |
| 25 | *S. aureus* 56400 | Blood | + | + | + | + | + | + | + | + | − |
| 26 | *S. aureus* 56142 | Blood | + | + | + | + | + | + | + | + | + |
| 27 | *S. aureus* 56266 | Blood | − | − | − | − | − | − | − | − | − |
| 28 | *S. aureus* 56223 | Blood | − | + | + | + | + | + | + | + | + |
| 29 | *S. aureus* 56686 | Blood | + | + | + | + | + | + | + | + | + |
| 30 | *S. aureus* 57259 | Blood | − | − | − | − | − | − | − | − | − |
| 31 | *S. aureus* 56555 | Blood | − | − | + | − | − | − | + | − | − |
| 32 | *S. aureus* 56568 | Blood | − | − | + | − | − | − | + | − | − |
| 33 | *S. aureus* 56618 | Blood | − | − | + | − | − | − | − | − | − |
| 34 | *S. aureus* A1244/19 | Blood | − | − | + | − | − | − | + | − | − |
| 35 | *S. aureus* A855/19 | Sputum | − | − | + | − | − | − | + | − | − |
| 36 | *S. aureus* A619/19 | Blood | − | − | + | − | − | − | + | − | − |
| 37 | *S. aureus* A1257/19 | Blood | − | − | + | − | − | − | − | − | − |
| 38 | *S. aureus* A1349/19 | Blood | − | − | + | − | − | − | + | − | − |
| 39 | *S. aureus* A193/19 | Sputum | − | − | + | − | − | − | + | − | − |
| 40 | *S. aureus* A192/19 | Urine | − | − | + | − | − | − | − | − | − |
| 41 | *S. aureus* A21/19 (61721) | Blood | − | − | + | − | − | − | + | − | − |
| 42 | *S. aureus* A22/19 (61722) | Pus | − | − | + | − | − | − | + | − | − |
| 43 | *S. aureus* A36/19 (61734) | Pus | − | − | + | − | − | − | + | − | − |
| 44 | *S. aureus* A513/19 | Wound | − | − | + | − | − | − | − | − | − |

| | | | | | | | | | | | |
|---|---|---|---|---|---|---|---|---|---|---|---|
| 45 | *S. aureus* A1277/19 | Tissue | − | − | + | − | − | − | − | − | − |
| 46 | *S. aureus* A1276/19 | Pus | − | − | + | − | − | − | + | − | − |
| 47 | *S. aureus* A1322/19 | Pus | − | − | + | − | − | − | + | − | − |
| 48 | *S. aureus* A53/19 (61747) | Pus | − | − | + | − | − | − | − | − | − |
| 49 | *S. aureus* A1348/19 | Sputum | − | − | + | − | − | − | − | − | − |
| 50 | *S. aureus* A618/19 | Blood | − | − | + | − | − | − | − | − | − |
| **Non-*Staphylococcus* strains** | | | | | | | | | | | |
| 1 | *Acinetobacter baumannii* ATCC 19606 | Culture collection (ATCC) | − | − | + | − | − | − | + | − | − |
| 2 | *Pseudomonas aeruginosa* ATCC 27853 | Culture collection (ATCC) | − | − | + | − | − | − | + | − | − |
| 3 | *Stenotrophomonas maltophilia* DMST 25614 | Culture collection (DMST) | − | − | + | − | − | − | + | − | − |
| 4 | *Salmonella Typhimurium* TISTR 2519 | Culture collection (TISTR) | − | − | + | − | − | − | + | − | − |
| 5 | *Escherichia coli* ATCC 25922 | Culture collection (ATCC) | − | − | + | − | − | − | + | − | − |
| 6 | *Bacillus cereus* ATCC 11778 | Culture collection (ATCC) | − | − | − | − | − | − | − | − | − |
| 7 | *Staphylococcus epidermidis* TISTR 518 | Culture collection (TISTR) | − | − | − | − | − | − | − | − | + |

**Note:** "positive" spot-test results indicate growth inhibition or clearing only and do not imply productive phage replication.
No plaque formation, efficiency-of-plating assays, burst size determination, or serial propagation was observed or performed on non-*Staphylococcus* hosts.

**Table S3**. Anti-biofilm activity of bacteriophages SA-W2 and MRSA-W3 against clinical and reference bacterial isolates
Biofilm biomass was quantified by crystal violet staining and measured as optical density at 595 nm. Values represent independent experimental replicates. Untreated wells served as controls.

| Isolate | SA-W2 Rep 1 | SA-W2 Rep 2 | SA-W2 Rep 3 | MRSA-W3 Rep 1 | MRSA-W3 Rep 2 | MRSA-W3 Rep 3 | Control Rep 1 | Control Rep 2 |
|---|---|---|---|---|---|---|---|---|
| 57711 | 0.118 | 0.123 | 0.122 | 0.127 | 0.120 | 0.129 | 0.131 | 0.126 |
| 57521 | 0.111 | 0.097 | 0.104 | 0.090 | 0.125 | 0.134 | 0.125 | 0.121 |
| 57263 | 0.120 | 0.104 | 0.113 | 0.110 | 0.122 | 0.121 | 0.118 | 0.144 |
| 57563 | 0.105 | 0.108 | 0.142 | 0.097 | 0.093 | 0.111 | 0.108 | 0.124 |
| 58775 | 0.105 | 0.104 | 0.108 | 0.094 | 0.098 | 0.114 | 0.111 | 0.120 |
| 58036 | 0.141 | 0.183 | 0.172 | 0.150 | 0.141 | 0.132 | 0.118 | 0.158 |
| 54760 | 0.106 | 0.113 | 0.148 | 0.109 | 0.112 | 0.112 | 0.128 | 0.130 |
| 57906 | 0.130 | 0.121 | 0.118 | 0.100 | 0.118 | 0.118 | 0.132 | 0.135 |
| 58736 | 0.120 | 0.131 | 0.139 | 0.110 | 0.133 | 0.113 | 0.122 | 0.140 |
| 56624 | 0.132 | 0.137 | 0.141 | 0.103 | 0.119 | 0.124 | 0.134 | 0.148 |
| 57235 | 0.111 | 0.165 | 0.129 | 0.123 | 0.139 | 0.128 | 0.144 | 0.140 |
| 57030 | 0.153 | 0.157 | 0.142 | 0.132 | 0.135 | 0.160 | 0.168 | 0.162 |
| 59037 | 0.143 | 0.164 | 0.203 | 0.182 | 0.168 | 0.168 | 0.159 | 0.620 |
| 58737 | 0.155 | 0.137 | 0.110 | 0.108 | 0.117 | 0.141 | 0.142 | 0.158 |
| 59762 | 0.160 | 0.176 | 0.148 | 0.138 | 0.151 | 0.189 | 0.166 | 0.110 |
| 58505 | 0.106 | 0.129 | 0.111 | 0.123 | 0.113 | 0.130 | 0.156 | 0.143 |
| 56488 | 0.152 | 0.141 | 0.141 | 0.120 | 0.134 | 0.153 | 0.129 | 0.141 |
| 55446 | 0.133 | 0.117 | 0.125 | 0.117 | 0.133 | 0.124 | 0.124 | 0.122 |
| 56686 | 0.144 | 0.124 | 0.106 | 0.108 | 0.109 | 0.120 | 0.117 | 0.126 |
| 57259 | 0.167 | 0.194 | 0.211 | 0.191 | 0.222 | 0.211 | 0.143 | 0.129 |
| 56223 | 0.146 | 0.142 | 0.110 | 0.116 | 0.178 | 0.139 | 0.120 | 0.114 |
| A53/1961717 | 0.133 | 0.162 | 0.125 | 0.125 | 0.133 | 0.134 | 0.122 | 0.136 |
| A1322/19 | 0.153 | 0.107 | 0.127 | 0.129 | 0.128 | 0.148 | 0.145 | 0.134 |
| 56568 | 0.179 | 0.166 | 0.173 | 0.117 | 0.159 | 0.152 | 0.145 | 0.192 |
| A36/19 61734 | 0.144 | 0.121 | 0.148 | 0.132 | 0.172 | 0.126 | 0.132 | 0.140 |
| A193/19 | 0.122 | 0.152 | 0.141 | 0.115 | 0.127 | 0.150 | 0.128 | 0.137 |
| A1276/19 | 0.129 | 0.110 | 0.094 | 0.093 | 0.124 | 0.120 | 0.121 | 0.143 |
| A1277/19 | 0.098 | 0.110 | 0.105 | 0.095 | 0.121 | 0.105 | 0.115 | 0.102 |
| 56618 | 0.131 | 0.132 | 0.112 | 0.097 | 0.102 | 0.131 | 0.126 | 0.124 |
| A855/19 | 0.121 | 0.122 | 0.103 | 0.095 | 0.097 | 0.110 | 0.110 | 0.110 |
| A21/19 61721 | 0.099 | 0.109 | 0.107 | 0.108 | 0.121 | 0.115 | 0.124 | 0.128 |
| A1349/19 | 0.098 | 0.105 | 0.105 | 0.113 | 0.104 | 0.130 | 0.112 | 0.110 |
| A1257/19 | 0.115 | 0.106 | 0.125 | 0.097 | 0.103 | 0.114 | 0.123 | 0.129 |
| A1348/19 | 0.123 | 0.125 | 0.118 | 0.108 | 0.112 | 0.130 | 0.117 | 0.115 |
| 58063 | 0.221 | 0.154 | 0.131 | 0.124 | 0.113 | 0.142 | 0.133 | 0.124 |
| *Samonella typhimurium* TISTR2519 | 0.187 | 0.151 | 0.135 | 0.130 | 0.128 | 0.122 | 0.149 | 0.192 |
| *E. coli* ATCC25922 | 0.144 | 0.139 | 0.139 | 0.139 | 0.134 | 0.147 | 0.125 | 0.149 |

| | | | | | | | | |
|---|---|---|---|---|---|---|---|---|
| *P. aeruginosa* ATCC27853 | 0.143 | 0.142 | 0.146 | 0.159 | 0.160 | 0.157 | 0.150 | 0.155 |
| *S. maltopholia* DMST25614 | 0.240 | 0.192 | 0.154 | 0.204 | 0.109 | 0.147 | 0.137 | 0.169 |
| *S. epidermidis* TISTB518 | 0.149 | 0.177 | 0.147 | 0.132 | 0.134 | 0.126 | 0.127 | 0.137 |
| *A. baumannii* ATCC19606 DMST 10437 | 0.228 | 0.225 | 0.206 | 0.197 | 0.170 | 0.177 | 0.196 | 0.156 |
| A618/19 | 0.191 | 0.129 | 0.156 | 0.139 | 0.120 | 0.139 | 0.150 | 0.128 |
| *B. cereus* ATCC11778 | 0.171 | 0.152 | 0.160 | 0.131 | 0.154 | 0.163 | 0.162 | 0.156 |
| A22/19 61722 | 0.141 | 0.144 | 0.121 | 0.108 | 0.109 | 0.120 | 0.119 | 0.110 |
| A11244/19 | 0.188 | 0.182 | 0.150 | 0.161 | 0.223 | 0.191 | 0.150 | 0.136 |
| A513/19 61747 | 0.167 | 0.173 | 0.110 | 0.156 | 0.155 | 0.144 | 0.128 | 0.111 |
| 56555 | 0.230 | 0.169 | 0.170 | 0.146 | 0.175 | 0.149 | 0.152 | 0.162 |
| A192/19 | 0.193 | 0.221 | 0.154 | 0.234 | 0.120 | 0.141 | 0.133 | 0.140 |
| A619/19 | 0.199 | 0.156 | 0.177 | 0.133 | 0.164 | 0.166 | 0.156 | 0.228 |
| 56752 | 0.171 | 0.158 | 0.167 | 0.115 | 0.128 | 0.130 | 0.139 | 0.105 |
| 56400 | 0.166 | 0.127 | 0.133 | 0.110 | 0.113 | 0.147 | 0.133 | 0.174 |
| 58037 | 0.127 | 0.123 | 0.114 | 0.101 | 0.122 | 0.121 | 0.119 | 0.139 |
| 56266 | 0.129 | 0.126 | 0.123 | 0.116 | 0.137 | 0.117 | 0.109 | 0.137 |
| 56142 | 0.129 | 0.128 | 0.134 | 0.126 | 0.140 | 0.130 | 0.121 | 0.130 |
| 58506 | 0.190 | 0.154 | 0.154 | 0.115 | 0.106 | 0.147 | 0.134 | 0.155 |
| 54784 | 0.123 | 0.135 | 0.147 | 0.112 | 0.122 | 0.134 | 0.118 | 0.108 |
| 58737 | 0.132 | 0.130 | 0.129 | 0.120 | 0.112 | 0.133 | 0.126 | 0.136 |
| 58747 | 0.135 | 0.126 | 0.108 | 0.171 | 0.123 | 0.142 | 0.117 | 0.131 |

**Notes:** Absorbance values reflect total biofilm biomass after crystal violet staining. No background subtraction or normalization was applied at this stage. These raw data were used to calculate percentage biofilm inhibition and 95% confidence intervals shown in Figure 2I. Non-*Staphylococcus* strains were included as specificity controls.

**Table S4a.** Effect of temperature and exposure time on the stability of bacteriophage MRSA-W3

| Temperature (°C) | Time (min) | $10^9$ PFU/mL | $10^8$ PFU/mL | $10^7$ PFU/mL | $10^6$ PFU/mL | $10^5$ PFU/mL | $10^4$ PFU/mL |
|---|---|---|---|---|---|---|---|
| **25** | 5 | P | P | P | 33 | 27 | 13 |
| | 30 | P | P | P | 55 | 18 | 9 |
| | 60 | P | P | P | 49 | 29 | 16 |
| | 120 | P | P | P | 55 | 33 | 14 |
| | 180 | P | P | P | 30 | 17 | 14 |
| **37** | 5 | P | P | P | P | P | P |
| | 30 | P | P | P | P | P | P |
| | 60 | P | P | P | P | P | P |
| | 120 | P | P | P | P | P | P |
| | 180 | P | P | P | P | P | P |
| **50** | 5 | P | P | P | P | P | P |
| | 30 | P | P | P | P | P | P |
| | 60 | P | P | P | P | P | P |
| | 120 | P | P | P | P | P | P |
| | 180 | P | P | P | P | P | P |
| **60** | 5 | P | P | P | P | P | P |
| | 30 | P | P | P | P | P | P |
| | 60 | P | P | P | P | P | P |
| | 120 | P | P | P | P | P | P |
| | 180 | P | P | P | P | P | P |

**Notes:** P = plaques observed (confluent lysis); numeric values indicate plaque counts.

**Table S4b.** Effect of temperature and exposure time on the stability of bacteriophage SA-W2

| **Temperature (°C)** | **Time (min)** | **$10^9$ PFU/mL** | **$10^8$ PFU/mL** | **$10^7$ PFU/mL** | **$10^6$ PFU/mL** | **$10^5$ PFU/mL** | **$10^4$ PFU/mL** |
|---|---|---|---|---|---|---|---|
| **25** | 5 | P | P | P | P | P | P |
| | 30 | P | P | P | P | P | P |
| | 60 | P | P | P | P | P | P |
| | 120 | P | P | P | P | P | P |
| | 180 | P | P | P | P | P | P |
| **37** | 5 | P | P | P | P | P | − |
| | 30 | P | P | P | P | P | − |
| | 60 | P | P | P | P | P | − |
| | 120 | P | P | P | P | P | − |
| | 180 | P | P | P | P | P | − |
| **50** | 5 | P | P | P | P | P | P |
| | 30 | P | P | P | P | P | P |
| | 60 | P | P | P | P | P | P |
| | 120 | P | P | P | P | P | P |
| | 180 | P | P | P | P | P | P |
| **60** | 5 | P | P | P | P | P | P |
| | 30 | P | P | P | P | P | P |
| | 60 | P | P | P | P | P | P |
| | 120 | P | P | P | P | P | + |
| | 180 | P | P | P | P | P | + |

**Notes:** P = plaques observed; (+) detectable plaques at the highest dilution; (−) no plaques observed.

**Table S5a.** Glass-based container surfaces disinfection efficacy of bacteriophage MRSA-W3 in organic solvent formulations

*Reduction of viable MRSA (DMST 20654) on container surfaces expressed as mean CFU/mL ± SD.*

| Treatment | PFU/mL | 2 min | 5 min | 10 min | 15 min | 20 min |
|---|---|---|---|---|---|---|
| MRSA-W3 + 1% Triton X-100 | $10^7$ | 289 ± 12.73 | >300 ± 0 | >300 ± 0 | >300 ± 0 | 289 ± 2.12 |
| | $10^6$ | 306 ± 0 | >300 ± 0 | >300 ± 0 | >300 ± 0 | 258 ± 14.85 |
| MRSA-W3 + 1% SDS | $10^7$ | 289 ± 30.41 | 278 ± 9.19 | 30 ± 0 | 215 ± 6.36 | 24 ± 11.31 |
| | $10^6$ | 2 ± 1.41 | 10 ± 9.90 | 69 ± 86.27 | 3 ± 2.12 | 0 ± 0 |
| MRSA-W3 + 1% Triton X-100 + 1% SDS | $10^7$ | 47 ± 2.83 | 12 ± 6.36 | 15 ± 8.49 | 2 ± 1.41 | 3 ± 2.83 |
| | $10^6$ | 50 ± 4.24 | 35 ± 7.07 | 24 ± 2.83 | 37 ± 36.77 | 6 ± 1.41 |
| MRSA-W3 + SM buffer | $10^7$ | 114 ± 0 | >300 ± 0 | >300 ± 0 | >300 ± 0 | 146 ± 0 |
| | $10^6$ | 166 ± 0 | >300 ± 0 | >300 ± 0 | >300 ± 0 | 150 ± 0 |
| 1% Triton X-100 (no phage) | – | >300 ± 0 | 20 ± 0 | 11 ± 0 | 0 ± 0 | 4 ± 0 |
| 1% SDS (no phage) | – | 0 ± 0 | >300 ± 0 | 1 ± 0 | 0 ± 0 | 2 ± 0 |
| MRSA control | – | >300 ± 0 | >300 ± 0 | >300 ± 0 | >300 ± 0 | 248 ± 0 |

**Table S5b. Surface disinfection efficacy of bacteriophage SA-W2 in organic solvent formulations**

*Reduction of viable S. aureus TISTR 746 on container surfaces expressed as mean CFU/mL ± SD.*

| Treatment | PFU/mL | 2 min | 5 min | 10 min | 15 min | 20 min |
|---|---|---|---|---|---|---|
| SA-W2 + 1% Triton X-100 | $10^7$ | 165 ± 4.95 | 49 ± 7.78 | 2 ± 0.71 | 3 ± 1.41 | 1 ± 0 |
| | $10^6$ | 88 ± 5.66 | 32 ± 0.71 | 82 ± 94.75 | 0 ± 0 | 1 ± 1.41 |
| SA-W2 + 1% SDS | $10^7$ | 170 ± 3.54 | 75 ± 29.70 | 7 ± 7.78 | 2 ± 2.12 | 0 ± 0 |
| | $10^6$ | 97 ± 21.21 | 91 ± 8.49 | 5 ± 2.83 | 6 ± 0 | 1 ± 1.41 |
| SA-W2 + 1% Triton X-100 + 1% SDS | $10^7$ | 71 ± 161.93 | 210 ± 63.64 | 7 ± 1.41 | 8 ± 0.71 | 3 ± 0.71 |
| | $10^6$ | 126 ± 12.73 | 115 ± – | 57 ± 8.49 | 3 ± 0 | 0 ± 0 |
| SA-W2 + SM buffer | $10^7$ | 161 ± 0 | 175 ± 0 | 162 ± 0 | 89 ± 0 | 50 ± 0 |
| | $10^6$ | 123 ± 0 | 34 ± 0 | 10 ± 0 | 24 ± 0 | 38 ± 0 |
| 1% Triton X-100 (no phage) | – | 60 ± 0 | 174 ± 0 | 11 ± 0 | 4 ± 0 | 0 ± 0 |
| 1% SDS (no phage) | – | 106 ± 0 | 25 ± 0 | 11 ± 0 | 0 ± 0 | 0 ± 0 |
| *S. aureus* control | – | 16 ± 0 | 41 ± 0 | 5 ± 0 | 0 ± 0 | 2 ± 0 |

**Notes:** Data are presented as mean ± SD (CFU/mL). >300 CFU/mL indicates confluent growth exceeding countable limits. Experiments were conducted on container surfaces under identical exposure conditions.

**Table S6.** Genome quality assessment and assembly statistics of *Staphylococcus aureus*-Infecting Phage MRSA-W3

*Assembly derived from bacteriophage-infected host culture; not virion-purified DNA*

| **Parameter** | **Value** |
|---|---|
| Genome assembly name | MRSA-W3_contigs.fa |
| Genome completeness (%) | 99.99 |
| Contamination (%) | 0.13 |
| Completeness model used | Neural network |
| Translation table | 11 (Bacterial, Archaeal, and Plant Plastid Code) |
| Coding density | 0.837 |
| Genome size (bp) | 2,745,360 |
| GC content (%) | 32.0 |
| Total coding sequences (CDSs) | 2,530 |
| Average gene length (bp) | 303.3 |
| Total number of contigs | 87 |
| N50 contig length (bp) | 186,171 |
| Maximum contig length (bp) | 459,358 |
| Additional notes | None |

**Note:** Genome completeness and contamination were estimated using a machine-learning–based model. Assembly statistics are reported for contigs ≥0 bp unless otherwise specified. Reported statistics refer to the infection-coupled assembly and include host-derived sequences; values should not be interpreted as representing a complete bacteriophage genome. **This assembly does not represent a complete bacteriophage genome.**

**Table S7a.** Integrated summary of prophage-like regions within assembled contigs identified in contig MRSA-W3_001

| Feature | Region 1 | Region 2 |
|---|---|---|
| Contig | MRSA-W3_001 | MRSA-W3_001 |
| Prophage-like regions within assembled contigs | Region 1 | Region 2 |
| Genomic position (bp) | 90,867–97,938 | 209,791–219,168 |
| Region length | ~7.0 kb | ~9.3 kb |
| Completeness | Incomplete | Incomplete |
| Prophage score | 20 | 20 |
| Total predicted proteins | 8 | 13 |
| Closest related phage | *Staphylococcus* phage SA97 (NC_029010) | *Staphylococcus* phage SPβ-like (NC_029119) |
| GC content (%) | 27.23 | 31.69 |
| Key phage-related features | Capsid and scaffold proteins, DNA-binding protein | Integrase, terminase, tail structure-related proteins |

**Note:** Both regions were classified as incomplete prophages but retained core phage-associated genes indicative of historical phage integration events.

**Table S7b.** Predicted coding sequences (CDSs) within prophage-like regions within assembled contigs 1 and 2 of contig MRSA-W3_001

**Region 1 (8 CDSs)**

| CDS position (bp) | Closest BLAST hit | Putative function | E-value |
|---|---|---|---|
| 90,867–91,064 | *Staphylococcus* phage vB_SauS_phi2 (NC_028862) | DNA-binding protein | $5.15 \times 10^{-9}$ |
| 91,769–91,981 | *Staphylococcus* phage StauST398_3 (NC_021332) | Hypothetical protein | $1.35 \times 10^{-9}$ |
| 92,276–92,482 | *Staphylococcus* phage phiMR11 (NC_010147) | Hypothetical protein | $3.22 \times 10^{-20}$ |
| 93,727–93,912 | *Staphylococcus* phage SA13 (NC_021863) | Hypothetical protein | $8.53 \times 10^{-28}$ |
| 94,424–94,840 | *Staphylococcus* phage 80 (NC_030652) | Hypothetical protein | $1.50 \times 10^{-25}$ |
| 95,678–97,027 | *Staphylococcus* phage StB27 (NC_019914) | Minor head protein | $3.63 \times 10^{-126}$ |
| 97,027–97,224 | No significant hit | Hypothetical protein | 0.0 |
| 97,384–97,938 | *Staphylococcus* phage StB27 (NC_019914) | Scaffold protein | $7.79 \times 10^{-10}$ |

**Region 2 (13 CDSs)**

| **CDS position (bp)** | **Closest BLAST hit** | **Putative function** | **E-value** |
|---|---|---|---|
| 209,791–211,281 (c) | *Escherichia* prophage MG1655 | Carboxy-terminal protease (PBP3-associated) | $1.48 \times 10^{-32}$ |
| 211,466–211,687 (c) | No significant hit | Hypothetical protein | 0.0 |
| 211,687–212,187 (c) | No significant hit | Hypothetical protein | 0.0 |
| 212,199–212,627 (c) | Phage phiNIH1.1 (NC_003157) | Methionine sulfoxide reductase | $3.20 \times 10^{-48}$ |
| 212,620–213,153 (c) | Phage phiNIH1.1 (NC_003157) | Methionine sulfoxide reductase | $1.99 \times 10^{-21}$ |
| 213,239–214,078 (c) | *Staphylococcus* phage SPβ-like (NC_029119) | DegV domain-containing protein | $1.13 \times 10^{-67}$ |
| 214,092–214,571 (c) | *Staphylococcus* phage SPβ-like (NC_029119) | Dihydrofolate reductase (DHFR) | $4.02 \times 10^{-92}$ |
| 214,770–215,726 (c) | *Staphylococcus* phage SPβ-like (NC_029119) | Thymidylate synthase | 0.0 |
| 216,146–216,583 (c) | No significant hit | Hypothetical protein | 0.0 |
| 216,600–217,346 (c) | No significant hit | Hypothetical protein | 0.0 |
| 217,350–217,724 (c) | No significant hit | Hypothetical protein | 0.0 |
| 217,762–218,013 (c) | No significant hit | Hypothetical protein | 0.0 |
| 218,464–219,168 | *Clostridium* phage CDKM15 (NC_048643) | Hypothetical protein | $5.21 \times 10^{-19}$ |

*(c) indicates CDS located on the complementary strand.*

**Table S8a. Summary of prophage-like regions within assembled contigs identified in contig MRSA-W3_007**

| **Parameter** | **Description** |
|---|---|
| Contig | MRSA-W3_007 |
| Prophage-like regions within assembled contigs | Region 1 |
| Genomic position (bp) | 62,402–68,309 |
| Region length | ~5.9 kb |
| Completeness | Incomplete |
| Prophage score | 20 |
| Total predicted proteins | 8 |
| Closest related phage | *Staphylococcus* phage phiSa119 (NC_025460) |
| GC content (%) | 29.77 |

**Note:** Prophage prediction was based on sequence similarity and phage hallmark genes.

**Table S8b.** Predicted coding sequences (CDSs) within prophage-like regions within assembled contigs 1 of contig MRSA-W3_007

| **CDS position (bp)** | **Closest BLAST hit** | **Putative function** | **E-value** |
|---|---|---|---|
| 62,402–62,992 | *Anoxybacillus* phage A403 (NC_048701) | Tail tip protein | $2.91 \times 10^{-8}$ |
| 63,075–64,070 | Deep-sea phage D6E (NC_019544) | Structural protein | $8.71 \times 10^{-29}$ |
| 64,146–64,772 | No significant hit | Hypothetical protein | 0.0 |
| 64,799–65,542 | *Staphylococcus* phage phiNM3 (NC_008617) | Enterotoxin type A precursor | $2.75 \times 10^{-30}$ |
| 65,569–66,321 | *Streptococcus* phage T12 (NC_028700) | Exotoxin type A | $4.47 \times 10^{-64}$ |
| 66,451–67,020 (c) | *Mycobacterium* phage Che9c (NC_004683) | gp32-like protein | $3.76 \times 10^{-7}$ |
| 67,205–67,651 (c) | No significant hit | Hypothetical protein | 0.0 |
| 68,073–68,309 (c) | *Paenibacillus* phage PBL1c (NC_048689) | Holin protein | $2.84 \times 10^{-27}$ |

*(c) indicates CDS located on the complementary strand.*

**Table S9a. Summary of prophage-like regions within assembled contigs identified in contig MRSA-W3_009**

| Parameter | Description |
|---|---|
| Contig | MRSA-W3_009 |
| Prophage-like regions within assembled contigs | Region 1 |
| Genomic position (bp) | 76,700–83,762 |
| Region length | ~7.0 kb |
| Completeness | Incomplete |
| Prophage score | 10 |
| Total predicted proteins | 7 |
| Closest related phage | Planktophage PaV-LD (NC_016564) |
| GC content (%) | 33.16 |

**Note:** Prophage prediction was based on sequence similarity and phage-related gene content.

**Table S9b.** Predicted coding sequences (CDSs) within prophage-like regions within assembled contigs 1 of contig MRSA-W3_009

| CDS position (bp) | Closest BLAST hit | Putative function | E-value |
|---|---|---|---|
| 76,700–76,948 (c) | *Enterococcus* phage IME_EFm1 (NC_024356) | Hypothetical protein | $7.31 \times 10^{-11}$ |
| 77,204–77,692 | Cyanophage RSM6 (NC_020855) | Peptide deformylase | $2.95 \times 10^{-17}$ |
| 77,685–78,620 | Prochlorococcus phage SSM7 (NC_015290) | PRGA-formyltransferase | $1.06 \times 10^{-13}$ |
| 78,617–79,924 | No significant hit | Hypothetical protein | 0.0 |
| 79,927–81,021 | *Pseudomonas* phage 201phi2-1 (NC_010821) | Fe–S cluster redox domain-containing protein | $4.06 \times 10^{-6}$ |
| 81,028–81,771 | Cronobacter phage phiES15 (NC_018454) | Putative serine/threonine phosphatase | $5.20 \times 10^{-5}$ |
| 81,768–83,762 | Shiga toxin-converting phage Stx2a (NC_049924) | Hypothetical protein | $5.05 \times 10^{-17}$ |

*(c) indicates CDS located on the complementary strand.*
